# Smad4 suppresses the tumorigenesis and aggressiveness of neuroblastoma through repressing the expression of heparanase

**DOI:** 10.1038/srep32628

**Published:** 2016-09-06

**Authors:** Hongxia Qu, Liduan Zheng, Wanju Jiao, Hong Mei, Dan Li, Huajie Song, Erhu Fang, Xiaojing Wang, Shiwang Li, Kai Huang, Qiangsong Tong

**Affiliations:** 1 Department of Pediatric Surgery, Union Hospital, Tongji Medical College, Huazhong University of Science and Technology, 1277 Jiefang Avenue, Wuhan 430022, Hubei Province, P. R. China; 2 Department of Pathology, Union Hospital, Tongji Medical College, Huazhong University of Science and Technology, 1277 Jiefang Avenue, Wuhan 430022, Hubei Province, P. R. China; 3 Clinical Center of Human Genomic Research, Union Hospital, Tongji Medical College, Huazhong University of Science and Technology, 1277 Jiefang Avenue, Wuhan 430022, Hubei Province, P. R. China

## Abstract

Heparanase (HPSE) is the only endo-β-D-glucuronidase that is correlated with the progression of neuroblastoma (NB), the most common extracranial malignancy in childhood. However, the mechanisms underlying HPSE expression in NB still remain largely unknown. Herein, through analyzing *cis*-regulatory elements and mining public microarray datasets, we identified SMAD family member 4 (Smad4) as a crucial transcription regulator of *HPSE* in NB. We demonstrated that Smad4 repressed the HPSE expression at the transcriptional levels in NB cells. Mechanistically, Smad4 suppressed the HPSE expression through directly binding to its promoter and repressing the lymphoid enhancer binding factor 1 (LEF1)-facilitated transcription of *HPSE* via physical interaction. Gain- and loss-of-function studies demonstrated that Smad4 inhibited the growth, invasion, metastasis, and angiogenesis of NB cells *in vitro* and *in vivo*. Restoration of HPSE expression prevented the NB cells from changes in these biological features induced by Smad4. In clinical NB specimens, Smad4 was under-expressed and inversely correlated with HPSE levels, while LEF1 was highly expressed and positively correlated with HPSE expression. Patients with high Smad4 expression, low LEF1 or HPSE levels had greater survival probability. These results demonstrate that Smad4 suppresses the tumorigenesis and aggressiveness of NB through repressing the HPSE expression.

Neuroblastoma (NB), a malignancy derived from neural crest cells of sympathetic nervous system, accounts for approximately 15% of all cancer-related mortality in childhood^1^. The clinical features of NB are heterogeneous, ranging from spontaneous regression to rapid progression and resistance to multimodal therapy^1^. For high-risk NB patients, tumor invasion and metastasis are the main causes of death, suggesting the urgency to investigate the underlying mechanisms for improving the outcome of NB patients^1^. It has been established that extracellular matrix (ECM) is an important structure surrounding the cells and vessels, and provides a physical barrier for the migration of tumor cells^2^. During the process of tumor invasion and metastasis, degradation of ECM and basement membrane is an initial and essential step^3^, which is also linked with tumor angiogenesis^3^. Thus, identification of crucial proteolytic enzymes that drive ECM degrading and remodeling will provide novel insights to improve the therapeutic efficiency of NB.

Heparanase (HPSE) is the only mammalian endo-β-D-glucuronidase that degrades the heparan sulphate glycosaminoglycan within the ECM and basement membrane^4^. In normal tissues, HPSE is detectable in platelets, neutrophils, and activated T lymphocytes 5. Meanwhile, up-regulation of HPSE has been demonstrated in a number of primary human malignancies, such as bladder cancer, prostate cancer, melanoma, pancreas cancer, colorectal cancer, and gallbladder cancer^6^, and is correlated with increased metastatic potentials and decreased survival rates^6^. It has also been documented that HPSE is functionally related to the invasion and metastasis of tumor cells^4^. In addition, HPSE is tightly involved in tumor angiogenesis by releasing angiogenic factors stored in the ECM such as basic fibroblast growth factor^7^, and promoting vascular endothelial growth factor (VEGF) expression via activation of the Src pathway^8^. Our previous studies have shown that HPSE is highly expressed in NB tissues, and is a prognostic factor for poor outcome of NB patients^9^. However, the mechanisms underlying the HPSE expression in NB still remain largely unknown.

In the current study, through analyzing the *cis*-regulatory elements and mining public microarray datasets, we identified SMAD family member 4 (Smad4) as a crucial transcription regulator of HPSE expression in NB. We demonstrate, for the first time, that Smad4 is under-expressed and inversely correlated with HPSE expression in clinical NB specimens. In addition, Smad4 represses the expression of HPSE through directly binding to its promoter and attenuating lymphoid enhancer binding factor 1 (LEF1)-facilitated transcription of *HPSE* via physical interaction, thus suppressing the growth, invasion, metastasis and angiogenesis of NB cells *in vitro* and *in vivo*, suggesting the tumor suppressive roles of Smad4 in the progression of NB.

## Results

### Smad4 represses the expression of HPSE in cultured NB cell lines

**Figure 1:**
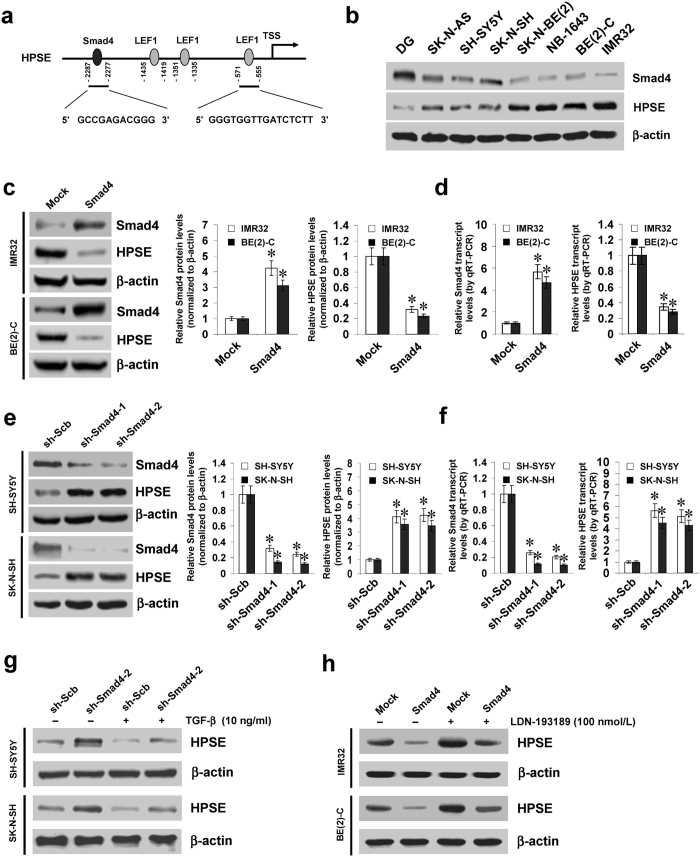
Smad4 represses the expression of HPSE in cultured NB cell lines. (**a)** scheme of the potential binding sites of Smad4 and LEF1 within *HPSE* promoter, locating at bases −2287/−2277, −1435/−1419, −1351/−1335, and −571/−555 upstream the transcription start site (TSS). (**b)** western blot showing the expression levels of Smad4 and HPSE in normal dorsal ganglia (DG) and NB cell lines. (**c**,**d**) western blot and real-time quantitative RT-PCR indicating the protein and transcript levels of Smad4 and HPSE in IMR32 and BE(2)-C cells stably transfected with empty vector (mock) or *Smad4*. (**e**,**f**) western blot and real-time quantitative RT-PCR showing the protein and transcript levels of Smad4 and HPSE in SH-SY5Y and SK-N-SH cells stably transfected with scramble shRNA (sh-Scb) or shRNA specific for Smad4 (sh-Smad4). (**g,h)** western blot indicating the expression levels of HPSE in NB cells stably transfected with sh-Scb, sh-Smad4, mock or *Smad4*, and those treated with TGF-β or LDN-193189. *P < 0.01 vs. mock or sh-Scb.

Mining the publicly available databases Genomatix^10^ and R2: microarray analysis and visualization platform (http://r2.amc.nl) revealed three potential transcription factors correlated with HPSE expression in NB tissues, including Smad4, LEF1, and peroxisome proliferator-activated receptor gamma (PPARG) (Supplementary Fig. S1a). However, transfection of short hairpin RNA (shRNA) specific for *PPARG* did not affect the *HPSE* promoter activity in NB cell lines (Supplementary Fig. S1b). Meanwhile, one Smad4 binding site and three LEF1 binding sites were noted within the *HPSE* promoter, locating at bases −2287/−2277, −1435/−1419, −1351/−1335, and −571/−555 relative to the transcription start site (TSS), respectively (Fig. 1a). Notably, negative or positive correlation between HPSE and Smad4 or LEF1 expression was observed in different NB and neuroblastic tumor cohorts (Supplementary Fig. S1c). Lower Smad4 and higher HPSE levels were observed in NB cell lines than those in normal dorsal ganglia (DG; Fig. 1b).

To investigate the effects of Smad4 on HPSE expression in NB cells, we performed the Smad4 over-expression and knockdown experiments. Western blot and real-time quantitative RT-PCR assays indicated that stable transfection of *Smad4* into IMR32 and BE(2)-C cells obviously increased the expression of Smad4, and decreased the HPSE levels, than those of empty vector (mock)-transfected cells (Fig. 1c,d). In contrast, transfection of shRNAs targeting Smad4 (sh-Smad4) into SH-SY5Y and SK-N-SH cells resulted in decreased protein and transcript levels of Smad4 and increased HPSE expression in NB cells, when compared to those stably transfected with scramble short hairpin RNA (sh-Scb) (Fig. 1e,f). Nuclear run-on assay indicated that stable over-expression or knockdown of *Smad4* decreased or increased the nascent transcript levels of *HPSE* in NB cells, respectively (Supplementary Fig. S2a). In SH-SY5Y and SK-N-SH cells, administration of transforming growth factor beta (TGF-β) resulted in increased activity of Smad4 response element reporter (Supplementary Fig. S2b) and decreased HPSE levels (Fig. 1g and Supplementary Fig. S2c), which were abolished by knockdown of *Smad4* (Fig. 1g, Supplementary Fig. S2b,c). However, treatment of IMR32 and BE(2)-C cells with either TGF-β or inhibitor of its receptors (LY364947)^11^, did not affect the nuclear translocation of Smad4 and inhibition of HPSE expression induced by stable transfection of *Smad4* (Supplementary Fig. S3a,b). Co-immunoprecipitation (Co-IP) and western blot assays indicated that transfection of D351H and R361H, two *Smad4* constructs with mutation of the loop-helix region^12^, abolished the interaction of Smad4 with R-Smads, phosphorylated Smad1 and Smad2 (p-Smad1 and p-Smad2), in these NB cells (Supplementary Fig. S3c). In addition, transfection of D351H or R361H did not influence the expression levels and promoter activity of *HPSE* (Supplementary Fig. S3d–f). Treatment with activin A-neutralizing antibody did not diminish the activation of Smad4 response element reporter in IMR32 and BE(2)-C cells stably transfected with *Smad4* (Supplementary Fig. S3g). Instead, administration of LDN-193189, the inhibitor of bone morphogenetic protein (BMP) type I receptors^13^, abolished the increased activity of BMP/Smad transcriptional reporter induced by BMP-2 (Supplementary Fig. S2h). Moreover, LDN-193189 treatment also prevented the IMR32 and BE(2)-C cells from decrease in the expression of *HPSE* induced by stable transfection of *Smad4* (Fig. 1h and Supplementary Fig. S3i). These results demonstrated that Smad4 considerably repressed the HPSE expression at the transcriptional levels in NB cells.

### Smad4 represses the transcription of *HPSE* through direct binding to its promoter

**Figure 2:**
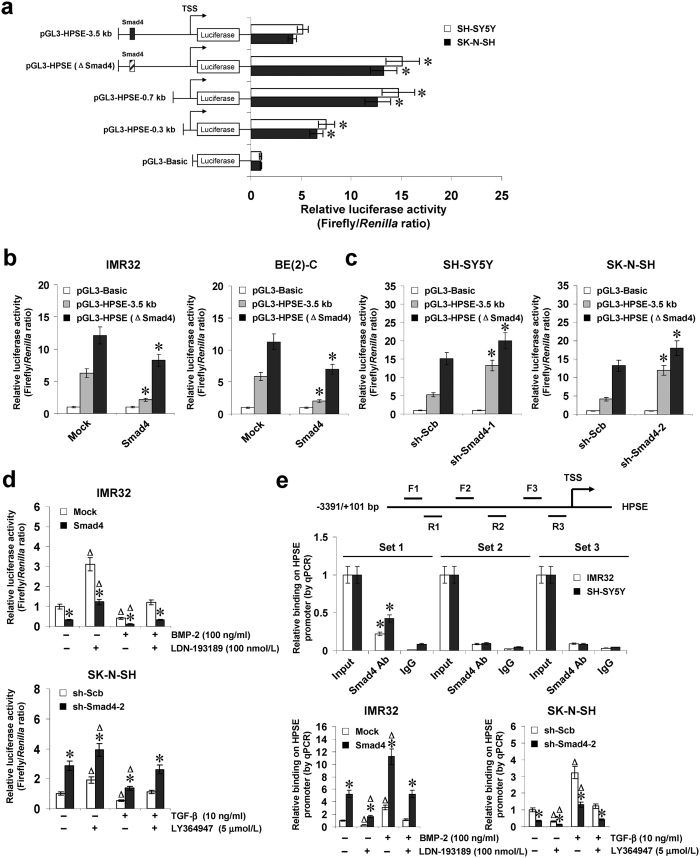
Smad4 represses the transcription of *HPSE* through direct binding to its promoter. (**a)** dual-luciferase assay showing the activity of *HPSE* promoter reporter and its truncates and mutant in SH-SY5Y and SK-N-SH cells. (**b**,**c)** dual-luciferase assay indicating the activity of *HPSE* promoter and its mutant in NB cells stably transfected with empty vector (mock), *Smad4*, scramble shRNA (sh-Scb), or shRNA specific for Smad4 (sh-Smad4). (**d)** dual-luciferase assay showing the activity of pGL3-HPSE-3.5 kb in IMR32 and SK-N-SH cells stably transfected with mock, *Smad4*, sh-Scb, or sh-Smad4, and those treated with BMP-2, LDN-193189, TGF-β protein, or LY364947. (**e)** ChIP and qPCR assay indicating the enrichment of Smad4 on *HPSE* promoter in IMR32 and SK-N-SH cells stably transfected with mock, *Smad4*, sh-Scb, or sh-Smad4, and those treated with BMP-2, LDN-193189, TGF-β protein, or LY364947. *P < 0.01 vs. pGL3-HPSE-3.5 kb, mock, sh-Scb, or IgG; ^Δ^*P* < 0.01 vs. control untreated with BMP-2, LDN-193189, TGF-β protein, or LY364947.

To determine whether Smad4 could directly target its binding site, the *HPSE* promoter luciferase reporter vector and its truncates were transfected into NB cells stably transfected with empty vector (mock) or *Smad4*. Dual-luciferase assay indicated that −3.5 to −0.7 kb relative to TSS was essential for the negative control of *HPSE* promoter activity, and mutation of Smad4 binding site within this region resulted in increased *HPSE* promoter activity in cultured SH-SY5Y and SK-N-SH cells (Fig. 2a). Ectopic expression or knockdown of *Smad4* attenuated and enhanced the promoter activity of *HPSE* in NB cells, respectively (Fig. 2b,c), and mutation of Smad4 binding site partially abolished these effects (Fig. 2b,c). In addition, BMP-2 treatment facilitated the decreased *HPSE* promoter activity in IMR32 cells stably transfected with *Smad4*, which was abolished by administration of LDN-193189 (Fig. 2d). Meanwhile, administration of TGF-β attenuated the increase in *HPSE* promoter activity induced by stable knockdown of *Smad4* in SK-N-SH cells, and these effects were abolished by LY364947 treatment (Fig. 2d). Chromatin immunoprecipitation (ChIP) and quantitative PCR (qPCR) assays were applied to measure the enrichment of Smad4 on *HPSE* promoter with three tiled primer sets. In cultured NB cells, enrichment of Smad4 was observed at the region (−2347/−2148) around its binding site (Fig. 2e). As controls, no *HPSE* promoter regions were immunoprecipitated with unspecific antibody (isotype IgG) (Fig. 2e). Stable transfection of *Smad4* or sh-Smad4 into IMR32 and SK-N-SH cells resulted in increased and decreased binding of Smad4 to *HPSE* promoter, respectively (Fig. 2e), which were abolished by treatment with LDN-193189 or TGF-β (Fig. 2e). These results indicated that Smad4 directly interacted with the binding site within *HPSE* promoter to repress its transcription.

### Smad4 represses the LEF1-facilitated transcription of *HPSE* in NB cells

**Figure 3:**
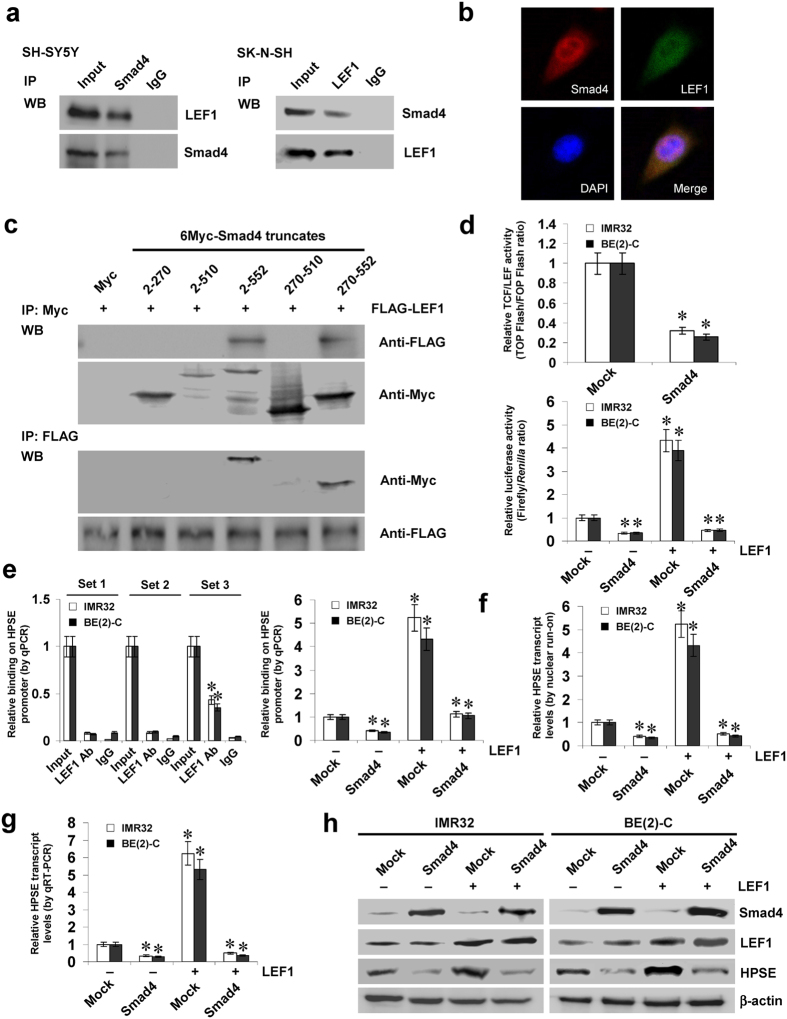
Smad4 represses the LEF1-facilitated transcription of *HPSE* in NB cells. (**a**,**b)** Co-IP and immunofluorescence assays revealing the endogenous interaction between Smad4 and LEF1 in NB cells. (**c)** IP and western blot assays showing the interaction between Smad4 and LEF1 in IMR32 cells transfected with different Myc-tagged *Smad4* truncates and FLAG-tagged *LEF1* construct, running under the same experimental conditions (full-length blots are presented in Supplementary Figure S7). (**d)** dual-luciferase assay indicating the activity of TCF/LEF-responsive constructs TOP-FLASH and FOP-FLASH and the *HPSE* promoter reporter in NB cells stably transfected with empty vector (mock) or *Smad4*, and those co-transfected with *LEF1*. (**e)** ChIP and qPCR assay showing the enrichment of LEF1 on *HPSE* promoter in NB cells stably transfected with mock or *Smad4*, and those co-transfected with *LEF1*. (**f**,**g)** nuclear run-on and real-time quantitative RT-PCR assays indicating the *HPSE* transcript levels in NB cells stably transfected with mock or *Smad4*, and those co-transfected with *LEF1*. (**h)** western blot assay showing the expression of Smad4, LEF1, and HPSE in NB stably transfected with mock or *Smad4*, and those co-transfected with *LEF1*. *P < 0.01 vs. mock or IgG.

Since previous studies indicate the interaction between Smad4 and LEF1^14^,^15^, and combining above evidence showing the potential roles of LEF1 in HPSE expression, we further investigated the effects of Smad4 on LEF1-mediated HPSE expression. As shown in Fig. 3a,b, Co-IP and immunofluorescence assays revealed the endogenous protein interaction between Smad4 and LEF1 in cultured NB cells. To determine the domain essential for this interaction, a series of Myc-tagged *Smad4* truncates were co-transfected with FLAG-tagged *LEF1* construct into NB cells, and the results indicated that the 511–552 amino acids of Smad4 was crucial for the interaction with LEF1 (Fig. 3c). Dual-luciferase assay indicated that stable transfection of *Smad4* attenuated the activity of T-cell factor (TCF)/LEF-responsive construct, and inhibited the LEF1-facilitated activity of *HPSE* promoter in IMR32 and BE(2)-C cells (Fig. 3d). ChIP and qPCR assays revealed the endogenous enrichment of LEF1 on the −673/−476 bp region, but not the −2347/−2148 or −1505/−1268 bp region, of *HPSE* promoter (Fig. 3e). Transfection of *LEF1* increased the *HPSE* promoter activity in NB cells, while mutation of LEF1 binding site within −673/−476 bp region abolished these effects (Supplementary Fig. S4a). In addition, transfection of *Smad4* prevented the increased enrichment of LEF1 on *HPSE* promoter induced by ectopic expression of *LEF1* (Fig. 3e). Dual-luciferase, nuclear run-on, real-time quantitative RT-PCR, and western blot assays further indicated that stable transfection of *Smad4* prevented the NB cells from increased promoter activity and expression levels of *HPSE* induced by LEF1 over-expression (Supplementary Fig. S4a, Fig. 3f–h). These results indicated that Smad4 repressed the LEF1-facilitated *HPSE* transcription in NB cells.

### Smad4 suppresses the growth, invasion, and angiogenesis of NB cells *in vitro*

**Figure 4:**
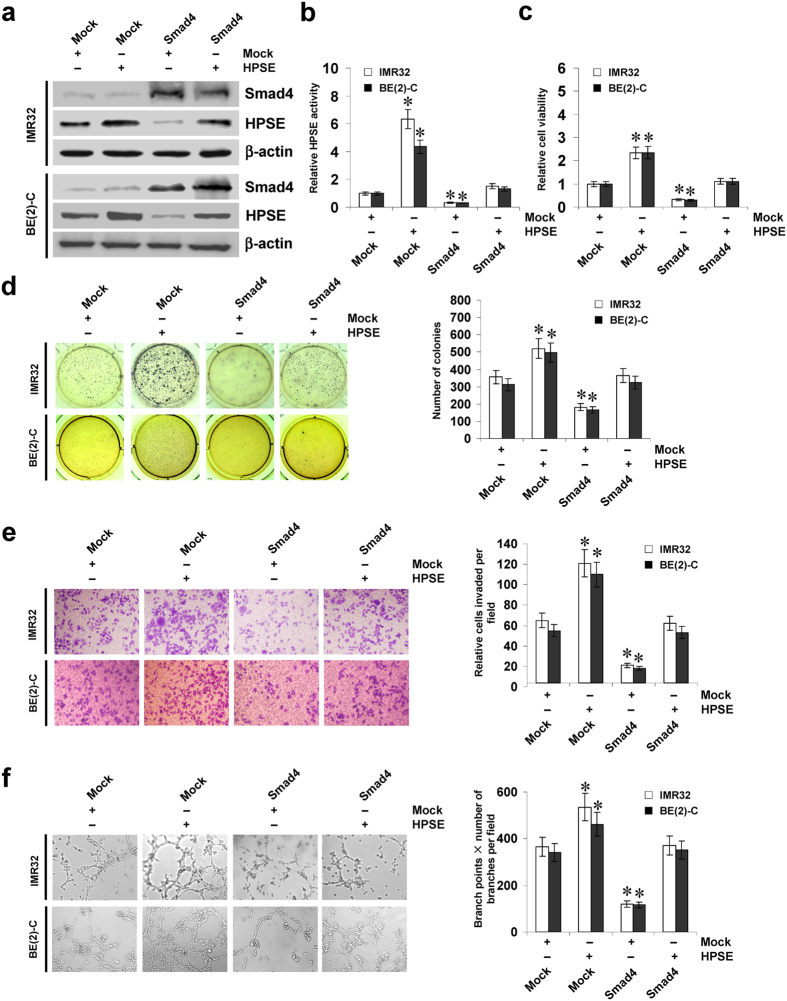
Smad4 suppresses the growth, invasion and angiogenesis of NB cells *in vitro*. (**a)** western blot indicating the expression of Smad4 and HPSE in IMR32 and BE(2)-C cells stably transfected with empty vector (mock) or *Smad4*, and those co-transfected with *HPSE*. (**b)** colorimetric assay for the HPSE activity in NB cells stably transfected with mock or *Smad4*, and those co-transfected with *HPSE*. (**c,d)** MTT colorimetric and soft agar assays [representation (left) and quantification (right)] showing the viability and anchorage-independent growth capability of NB cells stably transfected with mock or *Smad4*, and those co-transfected with *HPSE*. (**e)** representation (left) and quantification (right) of matrigel invasion assay indicating the invasion capability of NB cells transfected with mock or *Smad4*, and those co-transfected with *HPSE*. (**f)** representation (left) and quantification (right) of tube formation assay showing the angiogenic capability of NB cells transfected with mock or *Smad4*, and those co-transfected with *HPSE*. *P < 0.01 vs. mock.

Since previous studies indicate that HPSE participates in the growth, invasion, and angiogenesis of cancer cells^16^,^17^, and combining the evidence that Smad4 directly regulated the expression of HPSE, we investigated the effects of Smad4 over-expression and HPSE restoration on cultured NB cells. As shown in Fig. 4a,b, transfection of *HPSE* increased the expression and activity of HPSE, and restored the decrease in HPSE expression and activity induced by Smad4 in IMR32 and BE(2)-C cells. In MTT colorimetric and soft agar assays, stable transfection of *Smad4* suppressed the viability and anchorage-independent growth of NB cells, when compared to those stably transfected with empty vector (mock) (Fig. 4c,d). In matrigel invasion assay, Smad4 over-expression decreased the invasion capability of IMR32 and BE(2)-C cells (Fig. 4e). The tube formation of endothelial cells was decreased by treatment with the medium preconditioned by stable transfection of NB cells with *Smad4* (Fig. 4f). In addition, restoration of HPSE expression via transfection of *HPSE* vector prevented the NB cells from their decrease in growth, invasion, and angiogenesis induced by stable over-expression of *Smad4* (Fig. 4c–f).

On the other hand, we investigated the effects of Smad4 on LEF1-mediated HPSE activity, growth, invasion, and angiogenesis of NB cells. As shown in Supplementary Fig. S4b, increased HPSE activity was observed in NB cells stably transfected with *LEF1.* In MTT colorimetric and soft agar assays, LEF1 over-expression promoted the viability and anchorage-independent growth of IMR32 and BE(2)-C cells, when compared to those stably transfected with mock (Supplementary Fig. S4c,d). Matrigel invasion assay showed that NB cells stably transfected with *LEF1* presented an increased invasion capacity than mock-transfected cells (Supplementary Fig. S4e). The tube formation of endothelial cells was enhanced by treatment with the medium preconditioned by stable transfection of NB cells with *LEF1* (Supplementary Fig. S4f). In addition, transfection of *Smad4* rescued the IMR32 and BE(2)-C cells from their changes in HPSE activity, growth, invasion, and angiogenesis induced by ectopic expression of *LEF1* (Supplementary Fig. S4b–f). Collectively, these results revealed the tumor suppressive roles of Smad4 in regulating the growth, invasion, and angiogenesis of NB cells.

### Smad4 suppresses the growth, metastasis, and angiogenesis of NB cells *in vivo*

**Figure 5:**
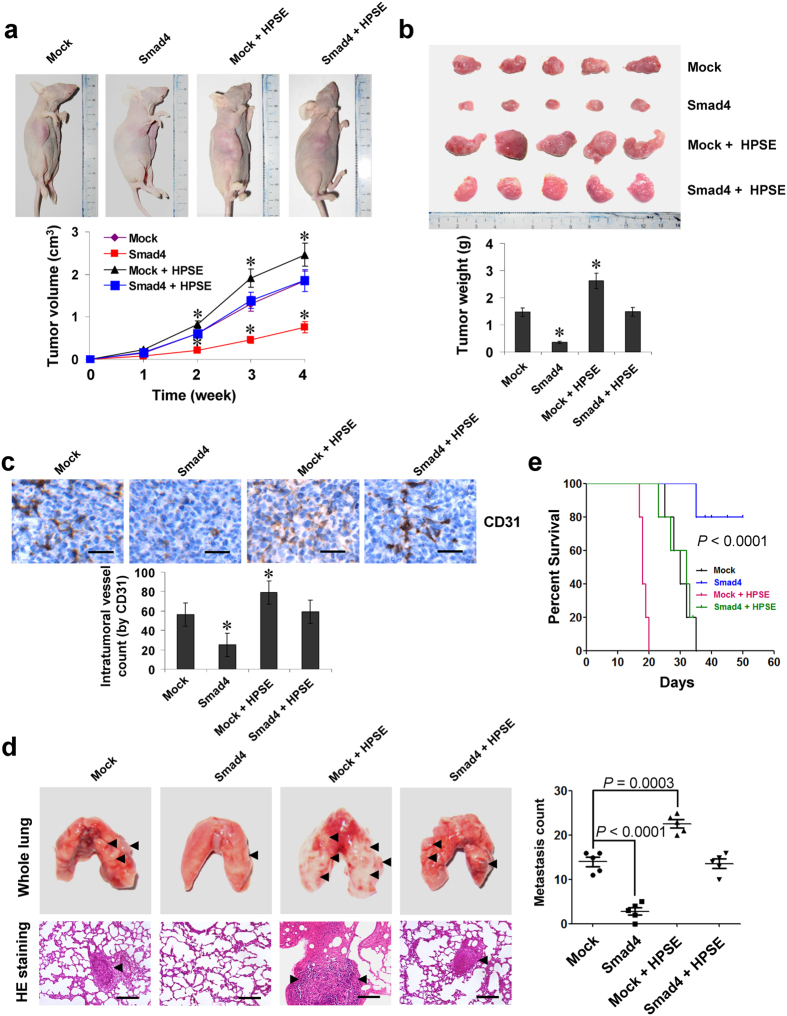
Smad4 suppresses the growth, metastasis and angiogenesis of NB cells *in vivo*. (**a)** tumor growth curve of BE(2)-C (1 × 10^6^) stably transfected with empty vector (mock) or *Smad4*, and those co-transfected with *HPSE* in athymic nude mice (n = 5 for each group), after hypodermic injection for 4 weeks. (**b)** representation (top) and quantification (bottom) of xenograft tumors formed by hypodermic injection of BE(2)-C cells stably transfected with mock or *Smad4*, and those co-transfected with *HPSE*. (**c)** immunohistochemical staining (top) and quantification (bottom) of CD31 expression within tumors formed by hypodermic injection of BE(2)-C cells stably transfected with mock or *Smad4*, and those co-transfected with *HPSE*. Scale bars: 100 μm. (**d)** representation (left, arrowhead) and quantification (right) of lung metastasis in nude mice with injection of BE(2)-C cells (0.4 × 10^6^) stably transfected with mock or *Smad4*, and those co-transfected with *HPSE* via the tail vein (n = 5 for each group). Scale bars: 100 μm. (**e)** Kaplan–Meier survival plots of athymic nude mice with injection of BE(2)-C cells (0.4 × 10^6^) stably transfected with mock or *Smad4*, and those co-transfected with *HPSE* via the tail vein (n = 5 for each group). *P < 0.01 vs. mock.

We next investigated the efficacy of Smad4 over-expression against tumor growth, metastasis, and angiogenesis *in vivo*. Stable transfection of *Smad4* into BE(2)-C cells resulted in decreased growth and tumor weight of subcutaneous xenograft tumors in athymic nude mice, when compared to those stably transfected with empty vector (mock; Fig. 5a,b). In addition, stable transfection of *Smad4* resulted in a decrease in CD31-positive mean vessel density within tumors (Fig. 5c). In the experimental metastasis studies, BE(2)-C cells stably transfected with *Smad4* established statistically fewer lung metastatic colonies and higher survival probability than mock group (Fig. 5d,e). Restoration of HPSE expression abolished these changes induced by stable over-expression of *Smad4* (Fig. 5a–e). These results were consistent with the findings that Smad4 suppressed the growth, invasion, and angiogenesis of NB cells *in vitro.*

### Smad4 and LEF1 are inversely or positively correlated with HPSE expression in NB tissues

**Figure 6:**
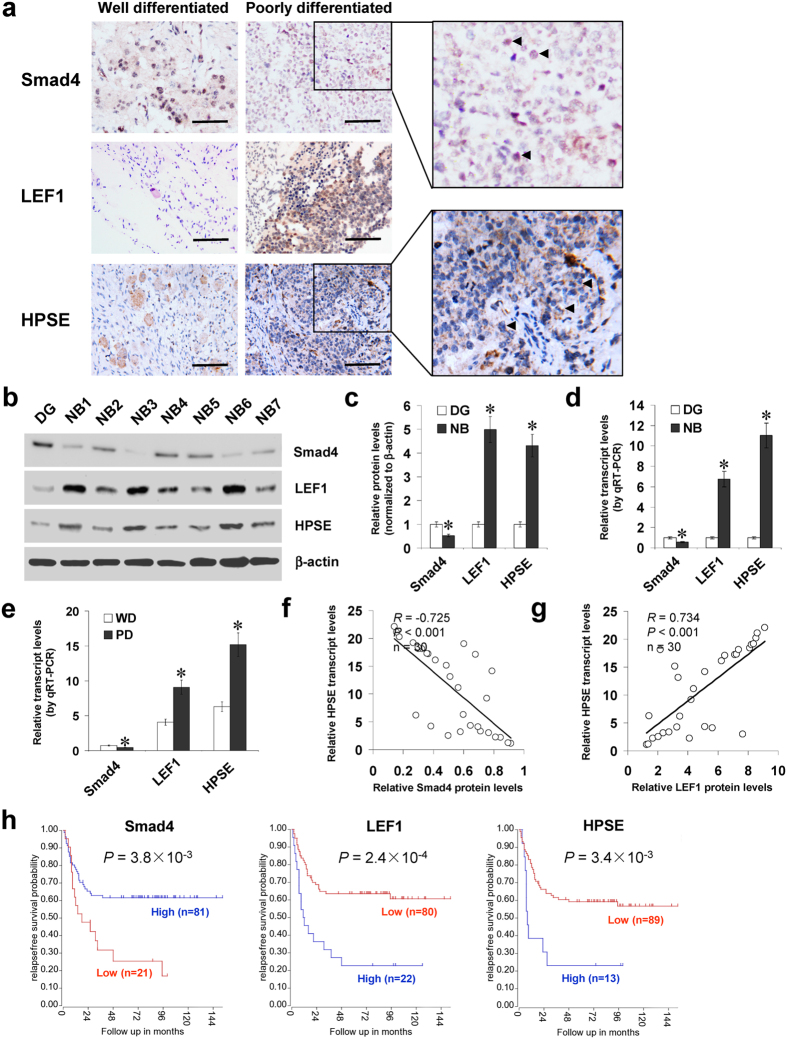
Smad4 and LEF1 are inversely or positively correlated with HPSE expression in NB tissues. (**a)** immunohistochemical staining revealing the expression of Smad4, LEF1, and HPSE in NB specimens (arrowheads, brown). Scale bars: 100 μm. (**b**–**d)** western blot and real-time quantitative RT-PCR assays showing the protein and transcript levels of Smad4, LEF1, and HPSE in NB tissues (n = 30) and normal dorsal ganglia (DG). (**e**) real-time quantitative RT-PCR indicating the transcript levels of *Smad4*, *LEF1*, and *HPSE* in NB tissues with well (WD) or poor differentiation (PD). (**f**–**g**) the correlation between Smad4 protein, LEF1 protein, and *HPSE* transcript levels in NB tissues (n = 30). (**h)** Kaplan–Meier survival plots of 102 well-defined NB cases derived from R2 microarray analysis and visualization platform (http://r2.amc.nl) showing the survival probability of patients with high or low expression of Smad4, LEF1 or HPSE. *P < 0.01 vs. DG or WD.

Mining the publicly available data derived from Oncogenomics (https://pob.abcc.ncifcrf. gov/cgi-bin/JK) and TARGET (https://target.nci.nih.gov/dataMatrix/) indicated no copy number loss and low mutation frequency of *Smad4* gene in NB (Supplementary Fig. S5a,b). To investigate the expression of Smad4 and LEF1 in NB tissues, paraffin-embedded sections from 42 well-established primary cases were collected. Immunohistochemical staining revealed that Smad4 and LEF1 were expressed in the nuclei of tumor cells (Fig. 6a). Smad4 expression was detected in 16/42 (38.1%) cases and the staining was weak in 8, moderate in 5, and intense in 3 (Supplementary Table S1). The Smad4 immunoreactivity was significantly lower in NB cases with poor differentiation (*P* < 0.001), higher mitosis karyorrhexis index (MKI) (*P* = 0.002), and advanced international neuroblastoma staging system (INSS) stages (*P* = 0.016) (Supplementary Table S1). Notably, the immunostaining of HPSE was inversely or positively correlated with that of Smad4 (correlation coefficient *R* = −0.583, *P* < 0.001) and LEF1 (correlation coefficient *R* = 0.553, *P* < 0.001) in NB cases, respectively (Fig. 6a and Supplementary Table S2). Western blot and real-time quantitative RT-PCR were applied to measure the expression levels of Smad4, LEF1, and HPSE in 30 NB specimens and DG. As shown in Fig. 6b–d, lower Smad4 expression and higher LEF1 or HPSE levels were observed in NB tissues than those in DG, which was in line with the data derived from R2: microarray analysis and visualization platform (Supplementary Fig. S5c). In addition, lower Smad4 levels and higher expression of LEF1 or HPSE were observed in NB specimens with poorer differentiation (Fig. 6e), advanced INSS stages (Supplementary Fig. S5d), or relapse (Supplementary Fig. S5d). There was an inverse correlation between Smad4 protein and *HPSE* transcript levels in NB tissues (correlation coefficient *R* = −0.725, *P* < 0.001, Fig. 6f), while LEF1 protein levels were positively correlated with *HPSE* transcript levels (correlation coefficient *R* = 0.734, *P* < 0.001, Fig. 6g). Kaplan–Meier survival plots of 102 well-defined NB cases derived from R2 microarray analysis and visualization platform revealed that patients with high Smad4 expression (*P* = 3.8 × 10^−3^), low LEF1 levels (*P* = 2.4 × 10^−4^), or low HPSE expression (*P* = 3.4 × 10^−3^) expression had greater survival probability (Fig. 6h), which was further validated by different NB cohorts derived from R2 database (Supplementary Fig. S6). These results indicated that Smad4 and LEF1 were inversely or positively correlated with HPSE expression in NB tissues.

## Discussion

*Smad4*, locating at chromosome 18q21, was first identified as a tumor suppressor gene in pancreatic cancer^18^. Subsequent studies show that Smad4 is involved in the TGF-β family signaling pathways^19^. The TGF-β family contains about 40 structurally related factors, including TGF-β, activin, inhibins, and BMPs, which exert signaling through binding to their receptors; upon receptor activation, the receptor-regulated Smads (R-Smads), including Smad1, Smad2, Smad3, Smad5 and Smad8, are phosphorylated to associate with the common Smad, Smad4, and mediate the nuclear translocation of heteromeric complex that regulates gene transcription^19^,^20^. It has been indicated that Smad4 is down-regulated in many human cancers, as a result of mutation, homozygous deletion, and heterozygous loss^21^. Mutation of *Smad4* gene occurs frequently in pancreatic^22^ and colorectal cancer^23^. Transfection of *Smad4* into pancreatic cancer cells reduces the anchorage-independent growth by more than 50%, and inhibits xenograft tumor growth^24^. In addition, stable over-expression of *Smad4* in colon cancer cells suppresses their growth *in vivo*^25^. However, few mutation of *Smad4* gene is seen in the rest of human cancers^26^. In the current study, we searched the publicly available databases Oncogenomics and TARGET, and found no copy number loss and low mutation frequency of *Smad4* in NB. We demonstrated the down-regulation of Smad4 in NB tissues and cell lines. Low Smad4 expression was associated with poor prognosis of NB patients, suggesting that Smad4 may be a potential outcome predictor for NB. In addition, we found that ectopic expression of *Smad4* suppressed the growth, invasion, metastasis, and angiogenesis of NB cells, suggesting the tumor suppressive roles of Smad4 in the progression of NB.

The heteromeric Smad complexes regulate target gene expression through direct binding to DNA, interaction with DNA-binding proteins, or recruitment of transcriptional co-activators or co-repressors^19^. Smad4 binds to the Smad-binding element, an 8-bp palindromic sequence (5′-GTCTAGAC-3′), for transcriptional regulation of TGF-β target genes^27^. Through targeting the promoter regions, Smad4 induces the expression of p15^ink4b^ to inhibit the growth of pancreatic cancer cells^24^. Enhanced Smad4 binding to the promoter of snail family zinc finger (*Snail*) contributes to its expression and epithelial-mesenchymal transition in skin carcinogenesis^28^. Over-expression of *Smad4* induces the expression of p21^waf1^ in breast cancer cells in the presence or absence of TGF-β stimulation, and Smad4 can bypass the TGF-β receptor activation to increase p21^waf1^ levels in pancreatic cancer cells^29^. In addition, Smad4 positively regulates the c-Myc expression in a TGF-β-independent manner^12^. In this study, we demonstrated that *HPSE* was a novel transcriptional target gene of Smad4. Previous studies have shown the existence of TGF-β/Smad signaling in some NB cell lines. TGF-β treatment facilitates the nuclear localization of Smad2 in SK-N-SH cells^30^. Ectopic over-expression of TGF-β type II receptor suppresses the tumor-forming ability and induces differentiation in LAN5 cells^31^. However, TGF-β and its receptors are usually down-regulated in aggressive or advanced stage NB^32^,^33^. Our findings indicated that TGF-β/Smad4 signaling was able to repress the HPSE expression in two non-*MYCN* amplified NB cells (SK-N-SH and SH-SY5Y), but not in *MYCN* amplified ones. In IMR32 and BE(2)-C cells, the Smad4-repressed HPSE expression was abolished by disruption of its interaction with p-Smad1 or p-Smad2, two R-Smads activated by BMP and activin receptors^19^,^20^. Since administration of activin A-neutralizing antibody did not affect the activity of Smad4 response element reporter in these cells, we ruled out the possible involvement of activin A signaling. Instead, as a member of BMP family expressing mainly in the nervous system, BMP-2 activated the BMP/Smad transcriptional reporter and facilitated the down-regulation of HPSE induced by Smad4 in IMR32 and BE(2)-C cells, which were abolished by the inhibitor of BMP type I receptors. It has been indicated that BMP-2 induces the phosphorylation of Smad1/5/8^34^, and is capable of facilitating growth arrest and neuronal differentiation in NB cells^35^. We believe that both TGF-β-dependent and -independent R-Smad activation occurs in NB cells to regulate the Smad4-mediated repression of HPSE expression in a context-dependent manner.

Human *HPSE* gene is located at chromosome 4q21.3, and its expression is regulated at the transcriptional levels^36^. The *HPSE* promoter contains GC-rich sequences and lacks a typical TATA or CCAAT box^36^. Previous studies indicate that transcription factors specific protein 1 and ETS are associated with basal *HPSE* promoter activity in thyroid tumor cells^37^, while early growth response 1 is implicated in inducible transcription of *HPSE* gene in prostate cancer cells^38^. In glioblastoma, glioma-associated oncogene homolog 1 facilitates the transcription of *HPSE* to promote tumor angiogenesis and aggressive growth^39^. Nuclear factor κB mediates the hypoxia-induced up-regulation of HPSE in pancreatic caner cells^40^. In addition, HPSE is induced in tumor cells by hepatocyte growth factor^41^, basic fibroblast growth factor^41^, and platelet-derived growth factor^41^. On the other hand, tumor suppressor p53 negatively regulates *HPSE* gene expression, suggesting that *p53* gene mutation may contribute to increased HPSE expression in tumors^42^. Despite extensive studies on the regulation of HPSE expression during tumorigenesis, little is known about *HPSE* gene transcription in NB. In this study, we demonstrated that LEF1 facilitated the *HPSE* transcription via directly binding to its promoter. Previous studies indicate that LEF1 forms a complex with Smad4 to activate the promoter of msh homeobox 2 in murine embryonic stem cells^15^. However, in palate medial-edge epithelial cells, LEF1 is activated by forming a complex with Smad4 to bind with the *E-cadherin* promoter to repress its transcription^14^. Our evidence shows that Smad4 is able to interact with LEF1 to abolish its binding to proximal *HPSE* promoter region. Over-expression of *Smad4* rescued the NB cells from LEF1-facilitated growth, invasion and angiogenesis, suggesting that Smad4 may exert its tumor suppressive functions, at least in part, through interacting and repressing the LEF1 activity in regulation of *HPSE* expression in NB.

In summary, we have shown that Smad4 is down-regulated in NB tissues, and inhibits the growth, invasion, metastasis, and angiogenesis of NB cells *in vitro* and *in vivo*. Furthermore, Smad4 suppresses the expression of HPSE via targeting its binding site within the promoter and attenuating LEF1-faclitated transcription in NB cell lines. This study extends our knowledge about the regulation of *HPSE* at the transcriptional level by transcription factors, and suggests that Smad4 and LEF1 may be of potential values as novel therapeutic targets for human NB. Meanwhile, the roles and underlying mechanisms of down-regulation of Smad4 in TGF-β family signaling pathways during the tumorigenesis and aggressiveness of NB warrant further investigation.

## Methods

### Cell culture

Human NB cell lines SK-N-AS (CRL-2137), SH-SY5Y (CRL-2266), SK-N-SH (HTB-11), SK-N-BE(2) (CRL-2271), NB-1643, BE(2)-C (CRL-2268), and IMR32 (CCL-127), and human endothelial cell line HUVEC (CRL-1730) were purchased from American Type Culture Collection (Rockville, MD) and Type Culture Collection of Chinese Academy of Sciences (Shanghai, China). Cell lines were authenticated by the provider, used within 6 months after resuscitation of frozen aliquots, and grown in RPMI1640 medium (Life Technologies, Inc., Gaithersburg, MD) supplemented with 10% fetal bovine serum (Life Technologies, Inc.), penicillin (100 U/ml) and streptomycin (100 μg/ml). Cells were maintained at 37 °C in a humidified atmosphere of 5% CO_2_, and applied for transfection or treatment with TGF-β protein, LY364947, BMP-2 protein, LDN-193189 (Sigma, St. Louis, MO), or activin A-neutralizing antibody (R&D Systems Inc., Minneapolis, MN) as indicated.

### Gene over-expression and knockdown

Human *Smad4* cDNA (1659 bp) was provided by Dr. Anna Coppa^43^ and subcloned into pcDNA3.1 (Invitrogen, Carlsbad, CA) with primer indicated in Supplementary Table S3. The 6Myc-tagged truncates and FLAG-tagged construct of *Smad4* were provided by Dr. Ralf Janknecht^44^ and Dr. Caroline S. Hill^45^, respectively. The Smad4 mutant constructs (D351H and R361H) were generated with GeneTailor^TM^ Site-Directed Mutagenesis System (Invitrogen) and PCR primers (Supplementary Table S3). Human *HPSE* cDNA (1632 bp) and *LEF1* cDNA (1200 bp) were amplified from NB tissue (Supplementary Table S3), and subcloned into pcDNA3.1 (Invitrogen) and pCMV-3Tag-1A (Addgene, Cambridge, MA), respectively. The empty vector (mock) and *Smad4* or *HPSE* constructs were transfected into tumor cells, and stable cell lines were screened by administration of neomycin or puromycin (Invitrogen). The mock-transfected cells were applied as controls. To restore the Smad4-induced down-regulation of *HPSE*, stable cell lines were transfected with the recombinant vector pcDNA3.1-HPSE or pCMV-3Tag-1A-LEF1. The oligonucleotides encoding shRNA specific for *Smad4* or *PPARG*, and their scramble sequences were subcloned into GV102 (Genechem Co., Ltd, Shanghai, China; Supplementary Table S3), and transfected into tumor cells with Genesilencer Transfection Reagent (Genlantis, San Diego, CA).

### Luciferase reporter assay

Human *HPSE* promoter luciferase reporter constructs were kindly provided by Dr. Xiulong Xu (Rush University Medical Center)^16^,^17^,^46^,^47^. The TCF/LEF-responsive reporter (TOP-FLASH and FOP-FLASH), Smad4 response element reporter (pSBE4-Luc), and BMP/Smad transcriptional reporter (pGL3-BRE-Luc) plasmids were obtained from Millipore (Billerica, MA) and Addgene, respectively. Mutation of Smad4 or LEF1 binding site was performed with GeneTailor^TM^ Site-Directed Mutagenesis System (Invitrogen) and PCR primers (Supplementary Table S3). Dual-luciferase assay was performed as previously described^16^,^17^,^46^,^47^.

### Nuclear run-on assay

Nuclear run-on assay was performed based on the incorporation of biotin-16-uridine- 5’-triphosphate into nascent transcripts as previously described^16^,^17^,^48^,^49^. Total RNA was extracted using Trizol (Invitrogen), and biotinylated nascent RNA was purified using agarose-conjugated streptavidin beads (Invitrogen). Beads were then eluted, and biotinylated RNA was isolated for real-time quantitative RT-PCR assay with primers (Supplementary Table S4).

### Western blot

Tissue or cellular protein was extracted with 1× cell lysis buffer (Promega, Madison, WI). Western blot was performed as previously described^47^,^50^,^51^,^52^,^53^, with antibodies specific for Smad4, p-Smad1, p-Smad2, LEF1, HPSE, H3 histone, and β-actin (Santa Cruz Biotechnology, Santa Cruz, CA). ECL substrate kit (Amersham, Piscataway, NJ) was used for the chemiluminscent detection of signals with autoradiography film (Amersham).

### Real-time quantitative RT-PCR

Total RNA was isolated with RNeasy Mini Kit (Qiagen Inc., Valencia, CA). The reverse transcription reactions were conducted with Transcriptor First Strand cDNA Synthesis Kit (Roche, Indianapolis, IN). Real-time PCR was performed with SYBR Green PCR Master Mix (Applied Biosystems, Foster City, CA) and primers indicated in Supplementary Table S4. The transcript levels were analyzed by 2^−△△Ct^ method.

### Co-immunoprecipitation

Co-IP was performed as previously described^16^, with antibodies specific for Smad4, LEF1, Myc, and FLAG (Upstate Biotechnology, Temacula, CA). The bead-bound proteins were released by boiling the protein A-Sepharose beads (Santa Cruz Biotechnology) in 1 × SDS-PAGE loading buffer and analyzed by western blot.

### Chromatin immunoprecipitation

ChIP assay was performed according to the manufacture’s instructions of EZ-ChIP kit (Upstate Biotechnology)^16^,^54^,^55^. DNA was sonicated into fragments of an average size of 200 bp. Different sets of PCR primers were designed, targeting the Smad4 and LEF1 binding site within *HPSE* promoter (Supplementary Table S4). Real-time qPCR with SYBR Green PCR Master Mix was performed using ABI Prism 7700 Sequence Detector.

### HPSE activity assay

The HPSE activity of tumor cells was measured according to the manufacture’s instructions of HepActiv^TM^ Heparanase Activity Assay Kit (InSight Biopharmaceuticals Ltd., Rehovot, Israel)^56^,^57^.

### Cell viability assay

Tumor cells were cultured in 96-well plates at 5 × 10^3^ cells per well. Cell viability was monitored by the 2-(4,5-dimethyltriazol-2-yl)-2,5-diphenyl tetrazolium bromide (MTT, Sigma) colorimetric assay^16^,^55^. All experiments were done with 6–8 wells per experiment and repeated at least three times.

### Soft agar assay

Tumor cells at 5 × 10^3^ were mixed with 0.05% Nobel agar (Fisher Scientific, Pittsburgh, PA) in growth medium and plated onto 6-well plates containing a solidified bottom layer (0.1% Noble agar in growth medium). After the incubation of cells for 21 days, the number of cell colonies was counted under the microscope, and the cells were fixed with 100% methanol and stained with 0.5% crystal violet dye^50^,^54^.

### Cell invasion assay

Matrigel invasion assay was performed using membranes coated with Matrigel matrix (BD Science, Sparks, MD). Homogeneous single cell suspensions (1 × 10^5^ cells/well) were added to the upper chambers and allowed to invade for 24 hrs at 37 °C in a CO_2_ incubator. Invaded cells were stained with 0.1% crystal violet for 10 min at room temperature and examined by light microscopy. Quantification of invaded cells was performed according to published criteria^47^,^50^,^51^,^52^,^55^,^58^.

### Tube formation assay

Fifty microliters of growth factor-reduced matrigel were polymerized on 96-well plates. HUVECs were serum starved in RPMI1640 medium for 24 hrs, suspended in RPMI1640 medium preconditioned with tumor cells, added to the matrigel-coated wells at the density of 5 × 10^4^ cells/well, and incubated at 37 °C for 18 hrs. Quantification of angiogenic activity was calculated by measuring the length of tube walls formed between discrete endothelial cells in each well relative to the control^17^,^48^,^49^,^51^,^55^.

### Immunofluorescence assay

Tumor cells were plated on coverslips, permeabilized with 0.3% Triton X-100, and blocked with 5% milk for 1 hr. Cells were incubated at 4 °C overnight with antibodies specific for Smad4 or LEF1 (Santa Cruz Biotechnology; 1:200 dilutions). Then, cells were incubated with Alexa Fluor 594 goat anti-rabbit IgG (1:1000 dilution), stained with 4′,6-diamidino-2-phenylindole (DAPI, 300 nmol/L) to visualize nuclei, and photographed under a microscope.

### *In vivo* growth, metastasis and angiogenesis assay

All animal experiments were carried out in accordance with NIH Guidelines for the Care and Use of Laboratory Animals, and approved by the Animal Care Committee of Tongji Medical College (approval number: Y20080290). For the *in vivo* tumor growth studies, 2-month-old male nude mice (n = 5 per group) were injected subcutaneously in the upper back with 1 × 10^6^ tumor cells stably transfected with empty vector, *Smad4* or *HPSE*. One month later, mice were sacrificed and examined for tumor weight and angiogenesis. The experimental metastasis (0.4 × 10^6^ tumor cells per mouse, n = 5 per group) studies were performed with 2-month-old male nude mice as previously described^17^,^47^,^48^,^49^,^50^,^54^,^58^.

### Patient tissue samples

Approval to conduct this study was obtained from the Institutional Review Board of Tongji Medical College (approval number: 2011-S085). All procedures were carried out in accordance with the approved guidelines. Paraffin-embedded specimens from 42 well-established primary NB cases were obtained from the Department of Pediatric Surgery, Union Hospital, Tongji Medical College^9^,^47^,^50^,^54^,^58^. Informed consent was obtained from all of the patients. The pathological diagnosis of NB was confirmed by at least two pathologists. Based on Shimada classification system, including the MKI, degree of neuroblastic differentiation and stromal maturation, and patient’s age, 19 patients were classified as favorable histology and 23 as unfavorable histology. According to the INSS, 7 patients were classified as stage 1, 7 as stage 2, 9 as stage 3, 11 as stage 4 and 8 as stage 4S. In subtotal 30 NB patients, fresh tumor specimens were collected at surgery and stored at −80 °C until use. Protein and RNAs of normal human dorsal ganglia were obtained from Clontech (Mountain View, CA).

### Immunohistochemistry

Immunohistochemical staining was performed as previously described^47^,^50^,^54^,^58^, with antibodies specific for Smad4, LEF1, HPSE (Abcam Inc, Cambridge, MA; Santa Cruz Biotechnology; 1:200 dilutions) and CD31 (Santa Cruz Biotechnology; 1:200 dilution). The negative controls included parallel sections treated with omission of the primary antibody, in addition to an adjacent section of the same block in which the primary antibody was replaced by rabbit polyclonal IgG (Abcam Inc.) as an isotype control. The immunoreactivity in each tissue section was assessed by at least two pathologists without knowledge of the clinicopathological features of tumors or patients’ survival. The degree of positivity was initially classified according to the percentage of positive tumor cells as the following: (−) <5% cells positive, (1+) 6–25% cells positive, (2+) 26–50% cells positive, and (3+) >50% cells positive.

### Statistical analysis

Unless otherwise stated, all data were shown as mean ± standard error of the mean (SEM). The χ^2^ analysis and Fisher exact probability analysis were applied for comparison among the gene expression and individual clinicopathological features. Pearson’s coefficient correlation was applied for analyzing the relationship among gene expression. The Kaplan-Meier method was used to estimate survival rates. Difference of tumor cells was determined by *t* test or analysis of variance (ANOVA).

## Additional Information

**How to cite this article**: Qu, H. *et al*. Smad4 suppresses the tumorigenesis and aggressiveness of neuroblastoma through repressing the expression of heparanase. *Sci. Rep.* **6**, 32628; doi: 10.1038/srep32628 (2016).

## Supplementary Material

Supplementary Information

## References

[b1] Westermann F. & Schwab M. Genetic parameters of neuroblastomas. Cancer Lett 184, 127–147 (2002). 12127685 10.1016/s0304-3835(02)00199-4

[b2] Yanagishita M. & Hascall V. C. Cell surface heparan sulfate proteoglycans. J Biol Chem 267, 9451–9454 (1992). 1577788

[b3] Liotta L. A., Steeg P. S. & Stetler-Stevenson W. G. Cancer metastasis and angiogenesis: An imbalance of positive and negative regulation. Cell 64, 327–336 (1991). 1703045 10.1016/0092-8674(91)90642-c

[b4] Hulett M. D. . Cloning of mammalian heparanase, an important enzyme in tumor invasion and metastasis. Nat Med 5, 803–809 (1999). 10395326 10.1038/10525

[b5] Nasser N. J. Heparanase involvement in physiology and disease. Cell Mol Life Sci 65, 1706–1715 (2008). 18425416 10.1007/s00018-008-7584-6 PMC11131613

[b6] Valentina M., Maria Francesca S., Giovanni G. & Maurizio O. Heparanase as a target in cancer therapy. Curr Cancer Drug Targets 14, 286–293 (2014). 24568197 10.2174/1568009614666140224155124

[b7] Elkin M. . Heparanase as mediator of angiogenesis: mode of action. FASEB J 15, 1661–1663 (2001). 11427519 10.1096/fj.00-0895fje

[b8] Zetser A. . Heparanase induces vascular endothelial growth factor expression: correlation with p38 phosphorylation levels and Src activation. Cancer Res 66, 1455–1463 (2006). 16452201 10.1158/0008-5472.CAN-05-1811

[b9] Zheng L. D. . Expression and clinical significance of heparanase in neuroblastoma. World J Pediatr 5, 206–210 (2009). 19693465 10.1007/s12519-009-0039-9

[b10] Cartharius K. . MatInspector and beyond: promoter analysis based on transcription factor binding sites. Bioinformatics 21, 2933–2942 (2005). 15860560 10.1093/bioinformatics/bti473

[b11] Shiou S. R. . Smad4 regulates claudin-1 expression in a transforming growth factor-beta-independent manner in colon cancer cells. Cancer Res 67, 1571–1579 (2007). 17308096 10.1158/0008-5472.CAN-06-1680 PMC6574217

[b12] Lim S. K. & Hoffmann F. M. Smad4 cooperates with lymphoid enhancer-binding factor 1/T cell-specific factor to increase c-myc expression in the absence of TGF-β signaling. Proc Natl Acad Sci USA 103, 18580–18585 (2006). 17132729 10.1073/pnas.0604773103 PMC1693705

[b13] Yu P. B. . BMP type I receptor inhibition reduces heterotopic ossification. Nat Med 14, 1363–1369 (2008). 19029982 10.1038/nm.1888 PMC2846458

[b14] Nawshad A., Medici D., Liu C. C. & Hay E. D. TGFβ3 inhibits E-cadherin gene expression in palate medial-edge epithelial cells through a Smad2-Smad4-LEF1 transcription complex. J Cell Sci 120, 1646–1653 (2007). 17452626 10.1242/jcs.003129 PMC2659570

[b15] Hussein S. M., Duff E. K. & Sirard C. Smad4 and beta-catenin co-activators functionally interact with lymphoid-enhancing factor to regulate graded expression of Msx2. J Biol Chem 278, 48805–48814 (2003). 14551209 10.1074/jbc.M305472200

[b16] Jiang G. . Small RNAs targeting transcription start site induce heparanase silencing through interference with transcription initiation in human cancer cells. PLoS One 7, e31379 (2012). 22363633 10.1371/journal.pone.0031379 PMC3282686

[b17] Qu H. . miRNA-558 promotes tumorigenesis and aggressiveness of neuroblastoma cells through activating the transcription of heparanase. Hum Mol Genet 24, 2539–2551 (2015). 25616966 10.1093/hmg/ddv018

[b18] Hahn S. A. . DPC4, a candidate tumor suppressor gene at human chromosome 18q21.1. Science 271, 350–353 (1996). 8553070 10.1126/science.271.5247.350

[b19] Massagué J. TGF-beta signal transduction. Annu Rev Biochem 67, 753–791 (1998). 9759503 10.1146/annurev.biochem.67.1.753

[b20] Katz L. H. . Targeting TGF-β signaling in cancer. Expert Opin Ther Targets 17, 743–760 (2013). 23651053 10.1517/14728222.2013.782287 PMC3745214

[b21] Yang G. & Yang X. Smad4-mediated TGF-β signaling in tumorigenesis. Int J Biol Sci 6, 1–8 (2010). 20087440 10.7150/ijbs.6.1 PMC2808050

[b22] Wilentz R. E. . Loss of expression of Dpc4 in pancreatic intraepithelial neoplasia: evidence that DPC4 inactivation occurs late in neoplastic progression. Cancer Res 60, 2002–2006 (2000). 10766191

[b23] Maitra A., Molberg K., Albores-Saavedra J. & Lindberg G. Loss of Dpc4 expression in colonic adenocarcinomas correlates with the presence of metastatic disease. Am J Pathol 157, 1105–1111 (2000). 11021814 10.1016/S0002-9440(10)64625-1 PMC1850169

[b24] Peng B. . Suppression of tumorigenesis and induction of p15(ink4b) by Smad4/DPC4 in human pancreatic cancer cells. Clin Cancer Res 8, 3628–3638 (2002). 12429655

[b25] Schwarte-Waldhoff I. & Schmiegel W. Smad4 transcriptional pathways and angiogenesis. Int J Gastrointest Cancer 31, 47–59 (2002). 12622415 10.1385/IJGC:31:1-3:47

[b26] Miyaki M. & Kuroki T. Role of Smad4 (DPC4) inactivation in human cancer. Biochem Biophys Res Commun 306, 799–804 (2003). 12821112 10.1016/s0006-291x(03)01066-0

[b27] Zawel L. . Human Smad3 and Smad4 are sequence-specific transcription activators. Mol Cell 1, 611–617 (1998). 9660945 10.1016/s1097-2765(00)80061-1

[b28] Hoot K. E. . Keratinocyte-specific Smad2 ablation results in increased epithelial- mesenchymal transition during skin cancer formation and progression. J Clin Invest 118, 2722–2732 (2008). 18618014 10.1172/JCI33713 PMC2447925

[b29] Hunt K. K. . Overexpression of the tumor suppressor gene Smad4/DPC4 induces p21waf1 expression and growth inhibition in human carcinoma cells. Cancer Res 58, 5656–5661 (1998). 9865717

[b30] Qiao J., Kang J., Ko T. C., Evers B. M. & Chung D. H. Inhibition of transforming growth factor-beta/Smad signaling by phosphatidylinositol 3-kinase pathway. Cancer Lett 242, 207–214 (2006). 16412560 10.1016/j.canlet.2005.11.007 PMC2614268

[b31] Turco A. . Increased TGFbeta type II receptor expression suppresses the malignant phenotype and induces differentiation of human neuroblastoma cells. Exp Cell Res 255, 77–85 (2000). 10666336 10.1006/excr.1999.4750

[b32] McCune B. K. . Expression of transforming growth factor-beta isoforms in small round cell tumors of childhood. An immunohistochemical study. Am J Pathol 142, 49–58 (1993). 8380955 PMC1886834

[b33] Iolascon A. . Reduced expression of transforming growth factor-beta receptor type III in high stage neuroblastomas. Br J Cancer 82, 1171–1176 (2000). 10735501 10.1054/bjoc.1999.1058 PMC2363349

[b34] Du Y. & Yip H. Effects of bone morphogenetic protein 2 on Id expression and neuroblastoma cell differentiation. Differentiation 79, 84–92 (2010). 19889495 10.1016/j.diff.2009.10.003

[b35] Nakamura Y., Ozaki T., Koseki H., Nakagawara A. & Sakiyama S. Accumulation of p27KIP1 is associated with BMP2-induced growth arrest and neuronal differentiation of human neuroblastoma-derived cell lines. Biochem Biophys Res Commun 307, 206–213 (2003). 12850001 10.1016/s0006-291x(03)01138-0

[b36] Dong J., Kukula A. K., Toyoshima M. & Nakajima M. Genomic organization and chromosome localization of the newly identified human heparanase gene. Gene 253, 171–178 (2000). 10940554 10.1016/s0378-1119(00)00251-1

[b37] Lu W. C., Liu Y. N., Kang B. B. & Chen J. H. Trans-activation of heparanase promoter by ETS transcription factors. Oncogene 22, 919–923 (2003). 12584571 10.1038/sj.onc.1206201

[b38] Ogishima T. . Increased heparanase expression is caused by promoter hypomethylation and up-regulation of transcriptional factor early growth response-1 in human prostate cancer. Clin Cancer Res 11, 1028–1036 (2005). 15709168

[b39] Zhu H., Carpenter R. L., Han W. & Lo H. W. The GLI1 splice variant TGLI1 promotes glioblastoma angiogenesis and growth. Cancer Lett 343, 51–61 (2014). 24045042 10.1016/j.canlet.2013.09.014 PMC3874262

[b40] Wu W. . Hypoxia activates heparanase expression in an NF-kappaB dependent manner. Oncol Rep 23, 255–261 (2010). 19956890

[b41] Sasaki M. . Erythromycin and clarithromycin modulation of growth factor-induced expression of heparanase mRNA on human lung cancer cells *in vitro*. Mediators Inflamm 10, 259–267 (2001). 11759110 10.1080/09629350120093731 PMC1781717

[b42] Baraz L., Haupt Y., Elkin M., Peretz T. & Vlodavsky I. Tumor suppressor p53 regulates heparanase gene expression. Oncogene 25, 3939–3947 (2006). 16474844 10.1038/sj.onc.1209425

[b43] D’Inzeo S. . A novel human Smad4 mutation is involved in papillary thyroid carcinoma progression. Endocr Relat Cancer 19, 39–55 (2012). 22109972 10.1530/ERC-11-0233

[b44] Janknecht R., Wells N. J. & Hunter T. TGF-β-stimulated cooperation of Smad proteins with the coactivators CBP/p300. Genes Dev 12, 2114–2119 (1998). 9679056 10.1101/gad.12.14.2114 PMC317012

[b45] Pierreux C. E., Nicolás F. J. & Hill C. S. Transforming growth factor beta-independent shuttling of Smad4 between the cytoplasm and nucleus. Mol Cell Biol 20, 9041–9054 (2000). 11074002 10.1128/mcb.20.23.9041-9054.2000 PMC86557

[b46] Li D. . Intelectin 1 suppresses the growth, invasion and metastasis of neuroblastoma cells through up-regulation of N-myc downstream regulated gene 2. Mol Cancer 14, 47 (2015). 25889839 10.1186/s12943-015-0320-6 PMC4359454

[b47] Zhao X. . CTCF cooperates with noncoding RNA MYCNOS to promote neuroblastoma progression through facilitating MYCN expression. Oncogene 35, 3565–3576 (2016). 26549029 10.1038/onc.2015.422

[b48] Xiang X. . miRNA-584-5p exerts tumor suppressive functions in human neuroblastoma through repressing transcription of matrix metalloproteinase 14. Biochim Biophys Acta 1852, 1743–1754 (2015). 26047679 10.1016/j.bbadis.2015.06.002

[b49] Xiang X. . miRNA-337-3p suppresses neuroblastoma progression by repressing the transcription of matrix metalloproteinase 14. Oncotarget 6, 22452–22466 (2015). 26084291 10.18632/oncotarget.4311 PMC4673175

[b50] Zhang H. . MicroRNA-145 inhibits the growth, invasion, metastasis and angiogenesis of neuroblastoma cells through targeting hypoxia-inducible factor 2 alpha. Oncogene 33, 387–397 (2014). 23222716 10.1038/onc.2012.574

[b51] Zheng L. . miRNA-145 targets v-ets erythroblastosis virus E26 oncogene homolog 1 to suppress the invasion, metastasis, and angiogenesis of gastric cancer cells. Mol Cancer Res 11, 182–193 (2013). 23233482 10.1158/1541-7786.MCR-12-0534

[b52] Zheng L. . microRNA-9 suppresses the proliferation, invasion and metastasis of gastric cancer cells through targeting cyclin D1 and Ets1. PLoS One 8, e55719 (2013). 23383271 10.1371/journal.pone.0055719 PMC3561302

[b53] Xiang X. . Hepatocyte nuclear factor 4 alpha promotes the invasion, metastasis and angiogenesis of neuroblastoma cells via targeting matrix metalloproteinase 14. Cancer Lett 359, 187–197 (2015). 25592038 10.1016/j.canlet.2015.01.008

[b54] Li D. . FOXD3 is a novel tumor suppressor that affects growth, invasion, metastasis and angiogenesis of neuroblastoma. Oncotarget 4, 2021–2044 (2013). 24269992 10.18632/oncotarget.1579 PMC3875767

[b55] Zheng L. . Methyl jasmonate abolishes the migration, invasion and angiogenesis of gastric cancer cells through down-regulation of matrix metalloproteinase 14. BMC Cancer 13, 74 (2013). 23394613 10.1186/1471-2407-13-74 PMC3576238

[b56] Hammond E., Li C. P. & Ferro V. Development of a colorimetric assay for heparanase activity suitable for kinetic analysis and inhibitor screening. Anal Biochem 396, 112–116 (2010). 19748475 10.1016/j.ab.2009.09.007

[b57] Nadanaka S., Purunomo E., Takeda N., Tamura J. & Kitagawa H. Heparan sulfate containing unsubstituted glucosamine residues: biosynthesis and heparanase-inhibitory activity. J Biol Chem 289, 15231–15243 (2014). 24753252 10.1074/jbc.M113.545343 PMC4140882

[b58] Zhang H. . microRNA-9 targets matrix metalloproteinase 14 to inhibit invasion, metastasis, and angiogenesis of neuroblastoma cells. Mol Cancer Ther 11, 1454–1466 (2012). 22564723 10.1158/1535-7163.MCT-12-0001

