# Supplementary material for: Smad4 suppresses the tumorigenesis and aggressiveness of neuroblastoma through repressing the expression of heparanase
Source: Sci Rep. 2016 Sep 6;6:32628. doi: 10.1038/srep32628 (PMC5011643; doi:10.1038/srep32628)
Supplement: Supplementary Information [file srep32628-s1.pdf]

Smad4 suppresses the tumorigenesis and aggressiveness of neuroblastoma through repressing the expression of heparanase

Hongxia Qu [1, #], Liduan Zheng [2, 3, #], Wanju Jiao [2, #], Hong Mei [1], Dan Li [1], Huajie Song [1], Erhu Fang [1], Xiaojing Wang [1], Shiwang Li [1], Kai Huang [3], and Qiangsong Tong [1, 3, *]

[1] Department of Pediatric Surgery, Union Hospital, Tongji Medical College, Huazhong University of Science and Technology, 1277 Jie Fang Avenue, Wuhan 430022, Hubei Province, P. R. China

[2] Department of Pathology, Union Hospital, Tongji Medical College, Huazhong University of Science and Technology, 1277 Jie Fang Avenue, Wuhan 430022, Hubei Province, P. R. China

[3] Clinical Center of Human Genomic Research, Union Hospital, Tongji Medical College, Huazhong University of Science and Technology, 1277 Jie Fang Avenue, Wuhan 430022, Hubei Province, P. R. China

Supplementary Figure Legends

Supplementary Figure S1 Mining the transcription factor crucial for HPSE expression. a, over-lapping analysis revealing Smad4, LEF1, and PPARG as potential transcription factors of HPSE in NB. **b**, real-time quantitative RT-PCR and dual-luciferase assays showing the *PPARG* levels and *HPSE* promoter activity in NB cells. **c**, the correlation between *Smad4*, *LEF1*, and *HPSE* levels in NB or neuroblastic tumor tissues derived from R2 microarray analysis and visualization platform (http://r2.amc.nl).

Supplementary Figure S2 Effects of TGF-β on HPSE expression in NB cells. a, nuclear run-on assay indicating the nascent transcript levels of *HPSE* in NB cells stably transfected with

mock, *Smad4*, sh-Scb, or sh-Smad4. **b**, dual-luciferase assay indicating the activity of Smad4 response element reporter (pSBE4-luc) and *HPSE* promoter reporter in NB cells stably transfected with sh-Scb or sh-Smad4, and those treated with TGF-β. * *P*<0.01 vs. mock or sh-Scb.

Supplementary Figure S3 Effects of BMP-2 on HPSE expression in NB cells. a and **b**, immunofluorescence and western blot assays indicating the localization of Smad4 and expression of HPSE in IMR32 and BE(2)-C cells stably transfected with mock or *Smad4*, and those treated with TGF-β or LY364947. **c**, Co-IP and western blot assays showing the interaction of Smad4 with p-Smad1 or p-Smad2 in IMR32 cells stably transfected with mock, *Smad4*, *Smad4 (D351H)*, or *Smad4 (R361H)*. **d**, **e**, and **f**, western blot, real-time quantitative RT-PCR, and dual-luciferase assays indicating the expression levels and promoter activity of *HPSE* in NB cells stably transfected with mock, *Smad4*, *Smad4 (D351H)*, or *Smad4 (R361H)*. **g**, **h**, and **i**, dual-luciferase and real-time quantitative RT-PCR assays showing the activity of Smad4 response element reporter (pSBE4-luc) or BMP/Smad transcriptional reporter (pGL3-BRE-Luc), and *HPSE* transcript levels in NB cells transfected with activin A-neutralizing antibody, BMP-2, or LDN-193189, and those transfected with mock or *Smad4*. * *P*<0.01 vs. mock; $^{\Delta}$*P*<0.01 vs. control untreated with BMP-2 or LDN-193189.

Supplementary Figure S4 Effects of Smad4 and LEF1 on the HPSE activity, growth, invasion, and angiogenesis of NB cells. Dual-luciferase (**a**), colorimetric (**b**), MTT colorimetric (**c**), soft agar (**d**), matrigel invasion (**e**), tube formation (**f**) assays showing the cellular *HPSE* promoter activity, HPSE enzymatic activity, viability, growth, invasion, and angiogenesis capability of NB cells stably transfected with empty vector (mock) or *Smad4*, and those

co-transfected with *LEF1*. * *P*<0.01 vs. mock.

Supplementary Figure S5 Expression of Smad4, LEF1, and HPSE in public databases. a and **b**, the datasets derived from Oncogenomics (https://pob.abcc.ncifcrf.gov/cgi-bin/JK) and TARGET (https://target.nci.nih.gov/dataMatrix/) indicating the copy number and mutation frequency of *Smad4* and *HPSE* in NB. **c** and **d**, the expression of *Smad4*, *LEF1*, and *HPSE* in NB tissues derived from R2 microarray analysis and visualization platform (http://r2.amc.nl).

Supplementary Figure S6 Patients' survival analysis. Kaplan–Meier survival plots of NB cases derived from R2 microarray analysis and visualization platform (http://r2.amc.nl) showing the survival probability of patients with high or low expression of Smad4 or HPSE.

Supplementary Figure S7 Interaction between Smad4 and LEF1 in NB cells. Co-IP and western blot assays showing the interaction between Smad4 and LEF1 in IMR32 cells transfected with different Myc-tagged *Smad4* truncates and FLAG-tagged *LEF1* construct.

Supplementary Figure S1

a

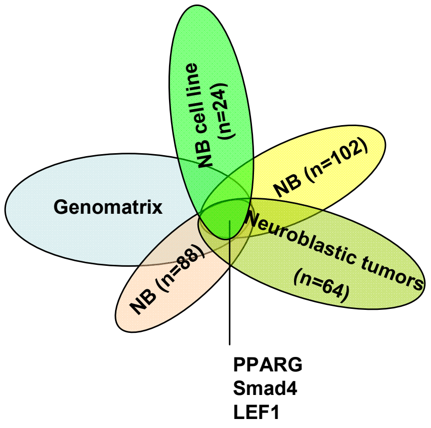

b

Correlation between PPARG and HPSE expression		
**R2 datasets**	***R*-value**	***P*-value**
NB (n=102)	0.314	0.001326016
NB (n=88)	0.249	0.019311638
Neuroblastic tumor (n=64)	0.411	0.000752421
NB cell lines (n=24)	0.487	0.015850924

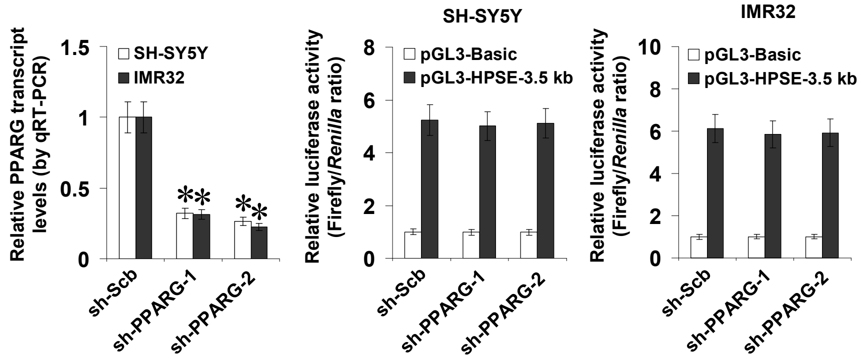

c

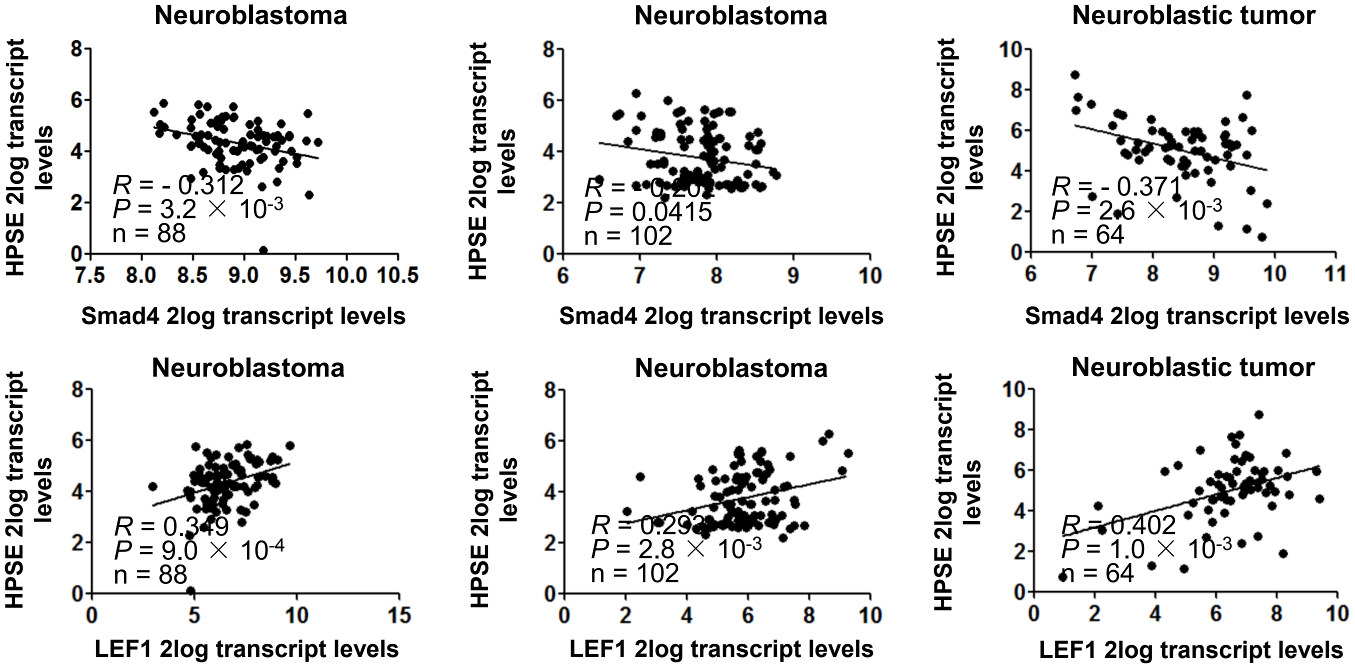

Supplementary Figure S2

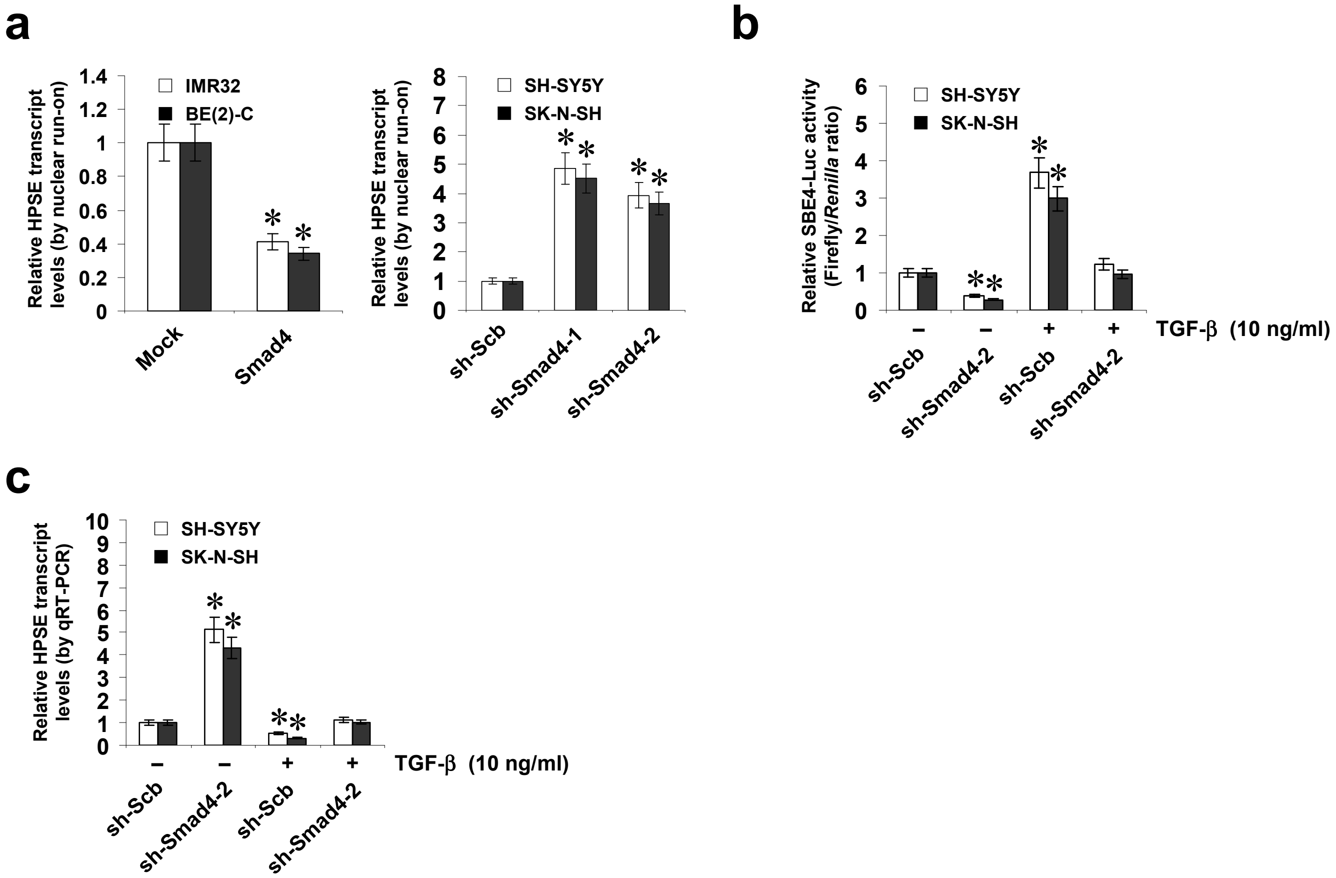

Supplementary Figure S3

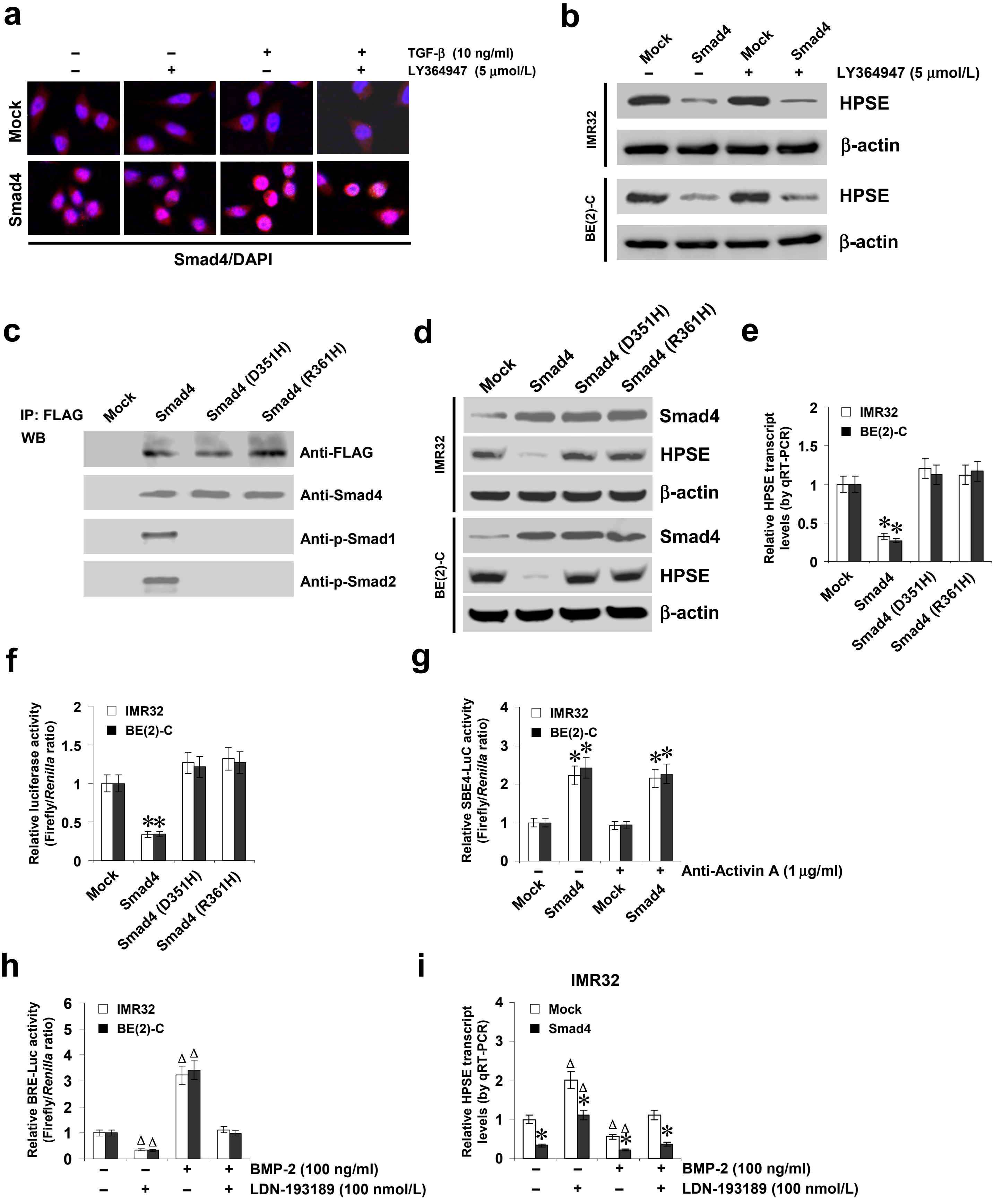

Supplementary Figure S4

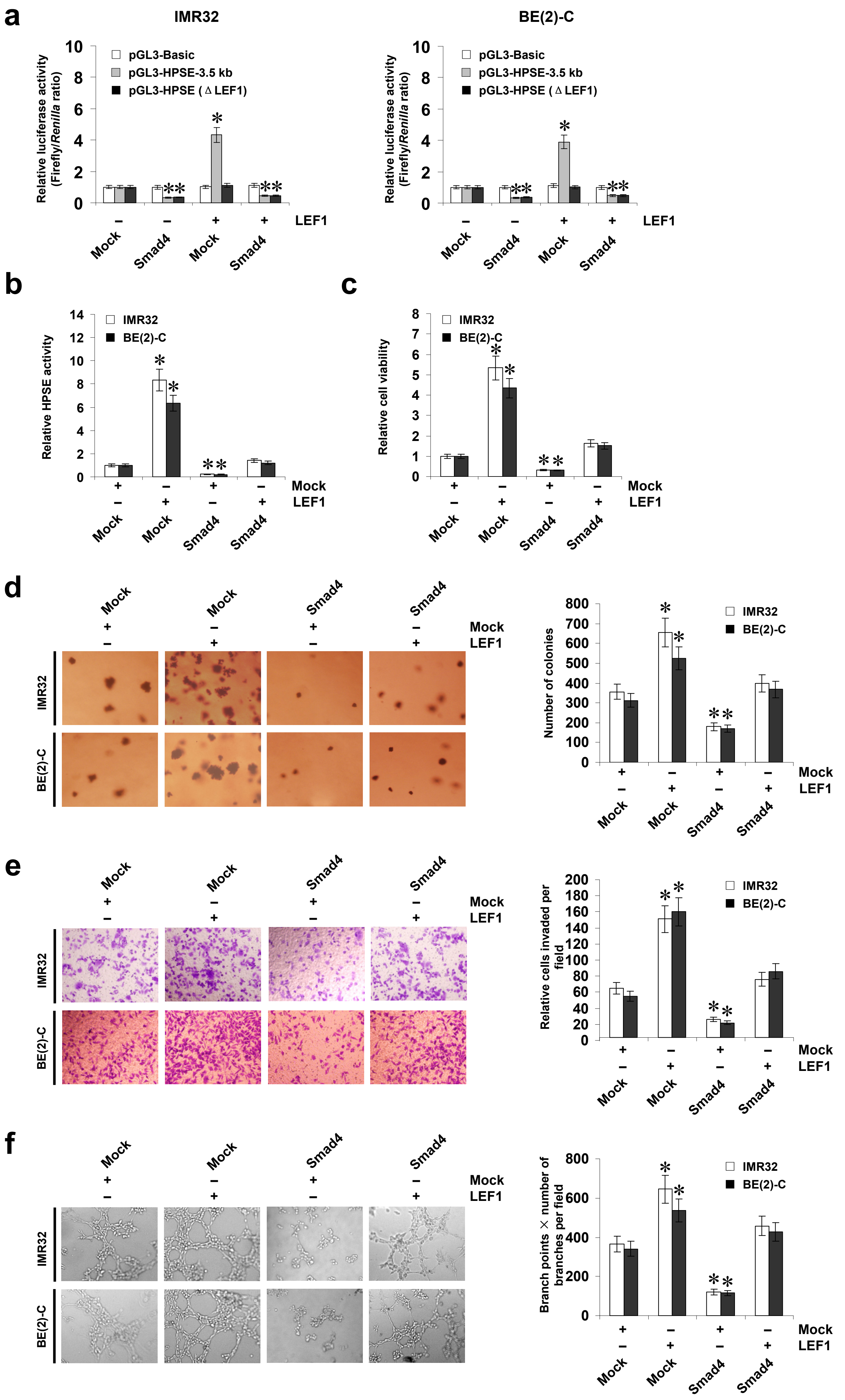

Supplementary Figure S5

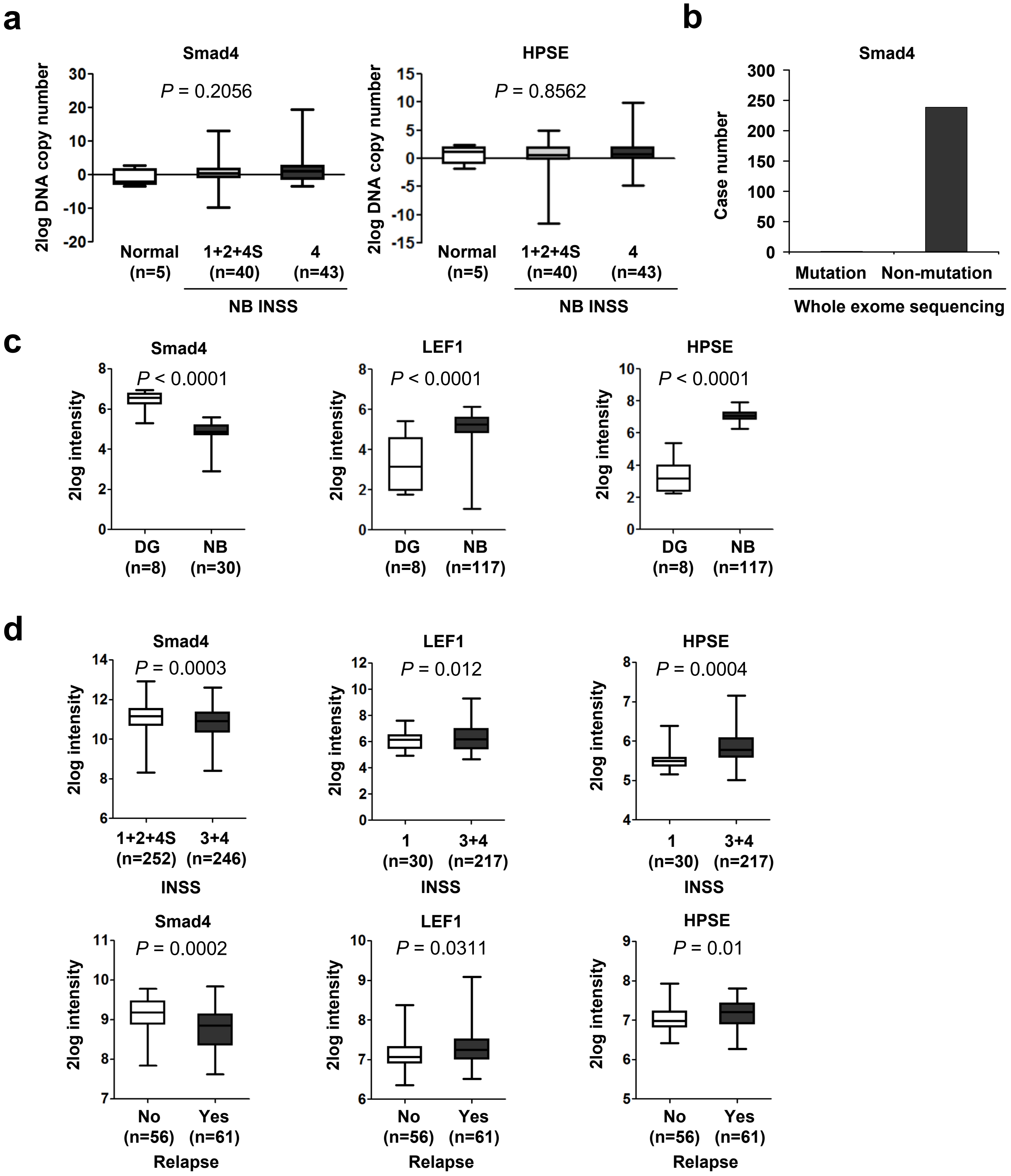

Supplementary Figure S6

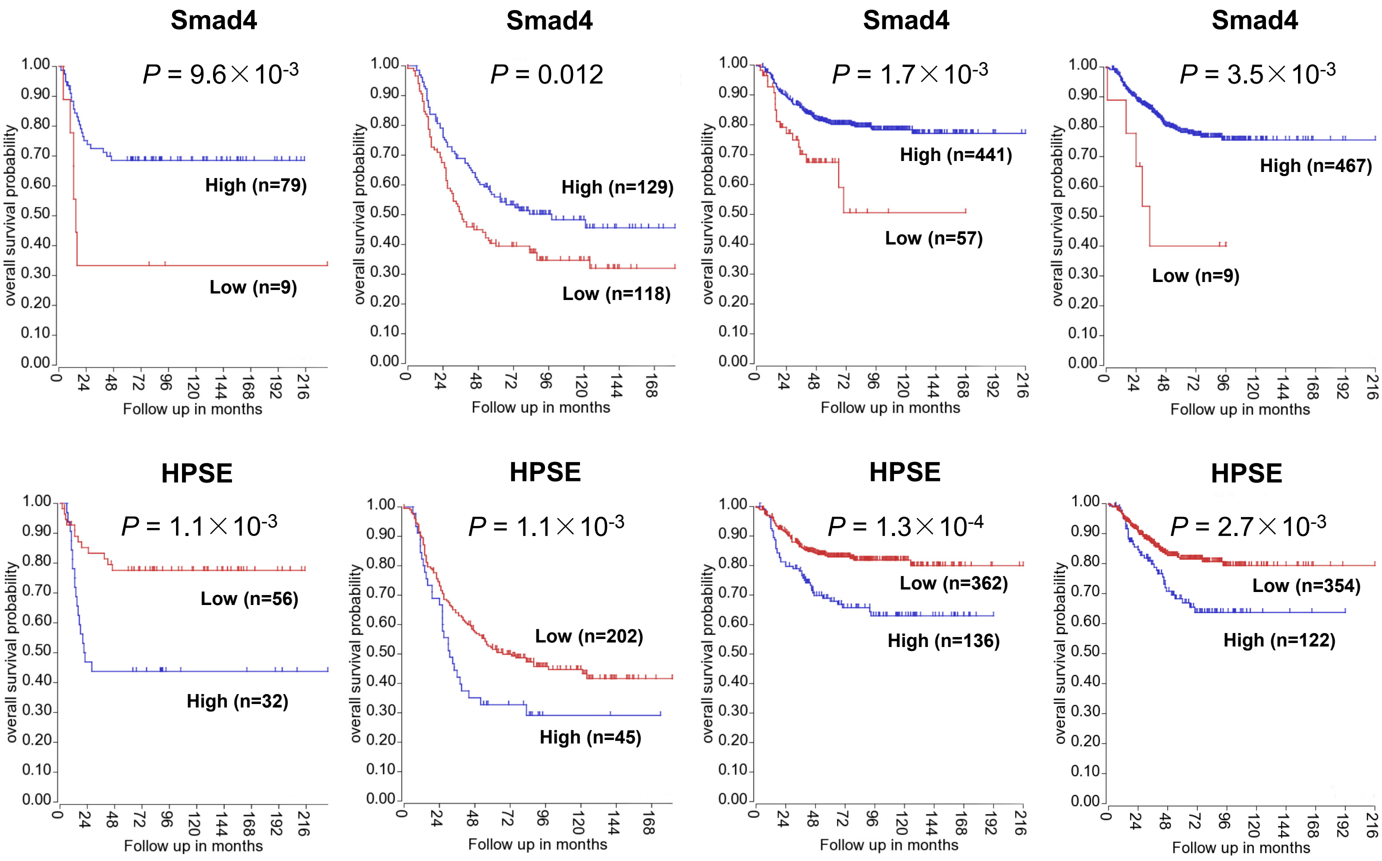

Supplementary Figure S7

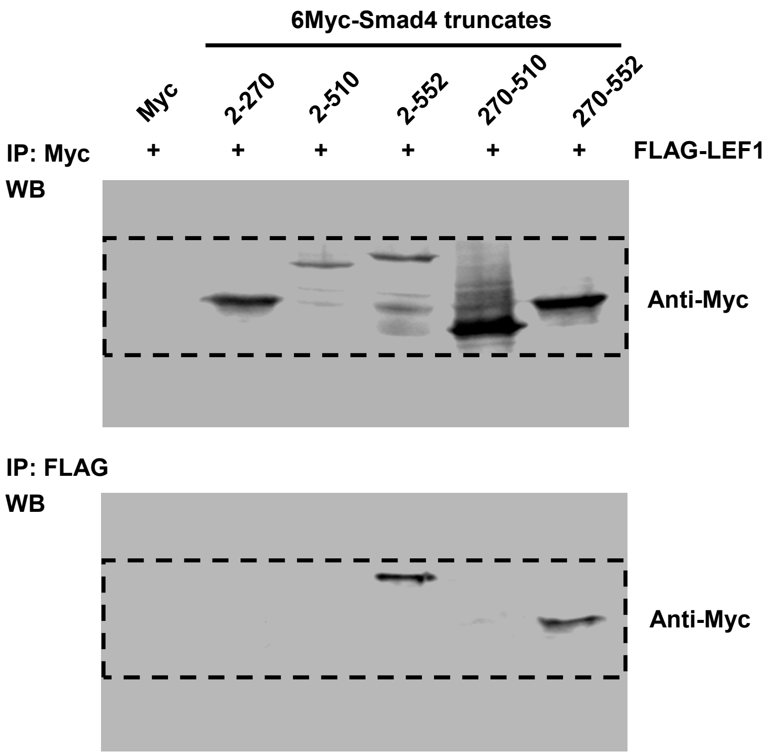

Supplementary Table S1 Smad4 expression in human NB tissues

Group	Total number	Smad4 expression				Positive rates (%)	*P*-Value
		−	+	++	+++		
Age							
<1 year	20	12	3	3	2	40.0	0.778
⩾1 year	22	14	5	2	1	36.4	
Differentiation							
Well differentiated	8	0	2	3	3	100.0	
Poorly differentiated	28	21	5	2	0	25.0	<0.001
Undifferentiated	6	5	1	0	0	16.7	
MKI							
<200	17	6	3	5	3	64.7	0.002
>200	25	20	5	0	0	20.0	
INSS stages							
Stage 1-2	14	4	3	4	3	71.4	
Stage 3-4	20	16	4	0	0	20.0	0.016
Stage 4S	8	6	1	1	0	25.0	

Smad4, SMAD family member 4; MKI, mitosis karyorrhexis index; INSS, international neuroblastoma staging system.

Supplementary Table S2 Correlation among the expression of Smad4, LEF1, and HPSE

	Smad4 expression				LEF1 expression			
	Low	High	*R*-value	*P*-value	Low	High	*R*-value	*P*-value
**HPSE expression**								
Low	4	6	- 0.583	<0.001	8	2	0.553	< 0.001
High	30	2			6	26		

Smad4, SMAD family member 4; LEF1, lymphoid enhancer-binding factor 1; HPSE, heparanase; Pearson's correlation coefficient was applied to determine the expression correlation.

Supplementary Table S3 Oligonucleotide sets used for constructs, inhibitors and short hairpin RNAs

Oligo Set	Sequences
pcDNA3.1-Smad4	5'-CGCGGATCCATGGACAATATGTCTATTACG-3' (sense);<br>5'-CCGCTCGAGTCAGTCTAAAGGTTGTGGGTC-3' (antisense)
pEF-FLAG-Smad4 (D351H)	5'-TGTTACTGTTCATGGATACGTGGACCCTTCTGGAGGAGATCG-3' (sense);<br>5'-CACGTATCCATGAACAGTAACAATAGGGCAGCTTGAAGGAAC-3' (antisense)
pEF-FLAG-Smad4 (R361H)	5'-GGAGGAGATCACTTTTGTTTGGGTCAACTCTCCAATGTCCAC-3' (sense);<br>5'-CCAAACAAAAGTGATCTCCTCCAGAAGGGTCCACGTATCCAT-3' (antisense)
pcDNA3.1-HPSE	5'-CGGGGTACCATGCTGCTGCGCTCGAAGCCTG-3' (sense);<br>5'-CCGCTCGAGTCAGATGCAAGCAGCAACTTTGG-3' (antisense)
pCMV-3Tag-1A-LEF1	5'-CGCGGATCCATGCCCCAACTCTCCGGAGGAGGT-3' (sense);<br>5'-CCCAAGCTTTCAGATGTAGGCAGCTGTCATTCT-3' (antisense)
pGL3-HPSE mut (∆ Smad4)	5'-TGTGAGGCCGCTGAGGGCAGATCGCGAGGTCAGGAGATTGAGACC-3' (sense);<br>5'-GCGATCTGCCCTCAGCGGCCTCACAAAATGCTAGGATTGCAGGCA-3' (antisense)
pGL3-HPSE mut (∆ LEF1)	5'-TTTGGGTGGCGACTCTCTTTCCAGCTGCAGTTTAGCGTATGCTG-3' (sense);<br>5'-CTGGAAAGAGAGTCGCCACCCAAAATTCTTTATCAACTTCTCATT-3' (antisense)
sh-Scb	5'-CCGGGCGAACGATCGAGTAAACGGACTCGAGTCCGTTTACTCGATCGTTCGCTTTTT-3' (sense);<br>5'-AATTCAAAAAGCGAACGATCGAGTAAACGGACTCGAGTCCGTTTACTCGATCGTTCGC-3' (antisense)
sh-Smad4-1	5'-CCGGGCAGACAGAAACTGGATTAAACTCGAGTTTAATCCAGTTTCTGTCTGCTTTTT-3' (sense);<br>5'-AATTCAAAAAGCAGACAGAAACTGGATTAAACTCGAGTTTAATCCAGTTTCTGTCTGC-3' (antisense)
sh-Smad4-2	5'-CCGGCCTGAGTATTGGTGTTCCATTCTCGAGAATGGAACACCAATACTCAGGTTTTT-3' (sense);<br>5'-AATTCAAAAACCTGAGTATTGGTGTTCCATTCTCGAGAATGGAACACCAATACTCAGG-3' (antisense)
sh-PPARG-1	5'-CCGGGCGCCTGCATCTCCACCTTATCTCGAGATAAGGTGGAGATGCAGGCGCTTTTT-3' (sense);<br>5'-AATTCAAAAAGCGCCTGCATCTCCACCTTATCTCGAGATAAGGTGGAGATGCAGGCGC-3' (antisense)
sh-PPARG-2	5'-CCGGGCGAAGCTTATCTATGACAGACTCGAGTCTGTCATAGATAAGCTTCGCTTTTT-3' (sense);<br>5'-AATTCAAAAAGCGAAGCTTATCTATGACAGACTCGAGTCTGTCATAGATAAGCTTCGC-3' (antisense)

Smad4, SMAD family member 4; HPSE, heparanase; LEF1, lymphoid enhancer-binding factor 1; sh-Scb, scramble short hairpin RNA; PPARG, peroxisome proliferator-activated receptor gamma.

Supplementary Table S4 Primer sets used for qRT-PCR, nuclear run-on, and ChIP

Primer set	Primers	Sequence	Product size (bp)	Application
HPSE	Forward	5'-GAATGGACGGACTGCTAC-3'	261	qRT-PCR,
	Reverse	5'-CCAAAGAATACTTGCCTCA-3'		nuclear run-on
Smad4	Forward	5'-TGCCTCACCACCAAAACGG-3'	255	qRT-PCR
	Reverse	5'-CCAAACAAAAGCGATCTCCTCC-3'		
LEF1	Forward	5'-AAATAAAGTGCCCGTGGTGC-3'	280	qRT-PCR
	Reverse	5'-TGAGGATGGGTAGGGTTGCC-3'		
PPARG	Forward	5'-GAACGACCAAGTAACTCTCCTC-3'	166	qRT-PCR
	Reverse	5'-GGGCTCCATAAAGTCACCAAAA-3'		
β-actin	Forward	5'-ATCTACGAGGGGTATGCC-3'	227	qRT-PCR
	Reverse	5'-TAGCTCTTCTCCAGGGAG-3'		
HPSE Set 1	Forward	5'-AAATTGGTATGACTGGGCATGG-3'	200	ChIP
(-2347/-2148)	Reverse	5'-TCAGCCTCCCGAGTAGCTGGGACTA-3'		
HPSE Set 2	Forward	5'-ACCTTTGGCCTCCCAAAATGCTGG-3'	238	ChIP
(-1505/-1268)	Reverse	5'-GGCATGTGATCATGTTGGTGCTTA-3'		
HPSE Set 3	Forward	5'-ACACTTTGTATGGGGGCTTCAG-3'	198	ChIP
(-673/-476)	Reverse	5'-CTCTGGCAAGGCAGTTTCTCAG-3'		

HPSE, heparanase; Smad4, SMAD family member 4; LEF1, lymphoid enhancer-binding factor 1; PPARG, peroxisome proliferator-activated receptor gamma.
